# Supplementary material for: Meta-analysis of effects of exclusive breastfeeding on infant gut microbiota across populations
Source: Nat Commun. 2018 Oct 9;9:4169. doi: 10.1038/s41467-018-06473-x (PMC6177445; doi:10.1038/s41467-018-06473-x)
Supplement: Supplementary file 1 — Supplementary Information [file 41467_2018_6473_MOESM1_ESM.pdf]

SUPPLEMENTARY INFORMATION

The effects of exclusive breastfeeding on the infant gut microbiota: a meta-analysis across populations

Ho et al.

List of supplementary Tables

Supplementary Table 1. List of shared genera of seven included studies used in the Random Forest models to predict gut microbiota age.

Supplementary Table 2. Meta-analysis of all seven included studies for gut bacterial taxa with differential relative abundances between non-exclusively breastfed vs. exclusively breastfed infants ≤6 months of age.

Supplementary Table 3. Meta-analysis of five studies that included a non-breastfeeding group for gut bacterial taxa with trend in relative abundance across exclusive breastfeeding, non-exclusive breastfeeding and non-breastfeeding groups.

Supplementary Table 4. Meta-analysis of six studies without data from North Carolina study for gut bacterial taxa with differential relative abundances between non-exclusively breastfed vs. exclusively breastfed infants ≤6 months of age (sensitivity analysis).

Supplementary Table 5. Meta-analysis of six studies without data from Haiti study for gut bacterial taxa with differential relative abundances between non-exclusively breastfed vs. exclusively breastfed infants ≤6 months of age (sensitivity analysis).

Supplementary Table 6. Meta-analysis of six studies without data from VDAART trial study for gut bacterial taxa with differential relative abundances between non-exclusively breastfed vs. exclusively breastfed infants ≤6 months of age (sensitivity analysis).

Supplementary Table 7. Meta-analysis stratified by mode of delivery for gut bacterial taxa with differential relative abundances between non-exclusively breastfed vs. exclusively breastfed infants ≤6 months of age.

Supplementary Table 8. Meta-analysis of all seven included studies for gut bacterial KEGG pathways with differential relative abundances between non-exclusively breastfed vs. exclusively breastfed infants ≤6 months of age.

Supplementary Table 9. Meta-analysis of included studies without data from either North Carolina, Haiti or VDAART trial study for gut bacterial KEGG pathways at level 3 with differential relative abundances between non-exclusively breastfed vs. exclusively breastfed infants ≤6 months of age (sensitivity analysis).

Supplementary Table 10. Meta-analysis stratified by mode of delivery for gut bacterial KEGG pathways with differential relative abundances between non-exclusively breastfed vs. exclusively breastfed infants ≤6 months of age.

Supplementary Table 11. Gut bacterial taxa with differential relative abundances from 6 months to 2 years of age between infants with duration of exclusive breastfeeding >2 months vs. ≤2 months from birth.

Supplementary Table 12. Breastfeeding is associated with reduced differences in gut bacterial taxa relative abundances between those who had vs. did not have diarrhea at the time of stool sample collection in infants from 6 months to 2 years of age.

Supplementary Table 13. Additional summary of included studies.

List of Supplementary Figures

Supplementary Figure 1. Meta-analysis of included studies without data from either North Carolina, Haiti or VDAART trial study for microbial alpha diversity difference between non-exclusively breastfed vs. exclusively breastfed infants (sensitivity analysis).

Supplementary Figure 2. Meta-analysis stratified by mode of delivery for differences in microbial alpha diversity (Shannon index) between non-exclusively breastfed vs. exclusively breastfed infants ≤6 months of age.

Supplementary Figure 3. Analysis adjusting for infant age vs. analysis adjusting for both infant age and sex for differences in microbial alpha diversity indexes between non-exclusively breastfed vs. exclusively breastfed infants ≤6 months of age.

Supplementary Figure 4. Performance of Random Forest model in prediction of gut microbiota age on the training and test set of Bangladesh data.

Supplementary Figure 5. Meta-analysis of included studies without data from either North Carolina, Haiti or VDAART trial study for microbiota age difference between non-exclusively breastfed vs. exclusively breastfed infants (sensitivity analysis).

Supplementary Figure 6. Meta-analysis stratified by mode of delivery for differences in microbiota age between non-exclusively breastfed vs. exclusively breastfed infants ≤6 months of age.

Supplementary Figure 7. Analysis adjusting for infant age vs. analysis adjusting for both infant age and sex for differences in microbiota age between non-exclusively breastfed vs. exclusively breastfed infants ≤6 months of age.

Supplementary Figure 8. Meta-analysis of seven included studies for the effects of non-exclusive vs. exclusive breastfeeding on relative abundance of gut bacterial taxa at order level in infants ≤ 6 months.

Supplementary Figure 9. Meta-analysis of seven included studies for the effects of non-exclusive vs. exclusive breastfeeding on relative abundance of gut bacterial taxa at genus level in infants ≤ 6 months.

Supplementary Figure 10. Meta-analysis stratified by mode of delivery for the effects of non-exclusive vs. exclusive breastfeeding on relative abundance of gut bacterial taxa at phylum level in infants ≤ 6 months.

Supplementary Figure 11. Meta-analysis stratified on vaginally delivered infants for the effects of non-exclusive vs. exclusive breastfeeding on relative abundance of gut bacterial taxa at family level in infants ≤ 6 months.

Supplementary Figure 12. Meta-analysis stratified on C-section delivered infants for the effects of non-exclusive vs. exclusive breastfeeding on relative abundance of gut bacterial taxa at family level in infants ≤ 6 months.

Supplementary Figure 13. Meta-analysis stratified on vaginally delivered infants for the effects of non-exclusive vs. exclusive breastfeeding on relative abundance of gut bacterial taxa at genus level in infants ≤ 6 months.

Supplementary Figure 14. Meta-analysis stratified on C-section delivered infants for the effects of non-exclusive vs. exclusive breastfeeding on relative abundance of gut bacterial taxa at genus level in infants ≤ 6 months.

Supplementary Figure 15. Analysis adjusting for infant age vs. analysis adjusting for both infant age and sex for the effects of non-exclusive vs. exclusive breastfeeding on relative abundance of gut bacterial taxa at phylum level in infants ≤ 6 months.

Supplementary Figure 16. Meta-analysis of seven included studies for the effects of non-exclusive vs. exclusive breastfeeding on relative abundances of gut microbial KEGG pathways at level two in infants ≤ 6 months.

Supplementary Figure 17. Analysis adjusting for infant age vs. analysis adjusting for both infant age and sex for the effects of non-exclusive vs. exclusive breastfeeding on relative abundances of gut microbial KEGG pathways at level three in infants ≤ 6 months.

Supplementary Figure 18. Longer duration of exclusive breastfeeding is associated with reduced effects of diarrhea at the time of stool sample collection on gut microbial diversity of infants from 6 months to 2 years of age.

List of Supplementary Notes

Supplementary Note 1. List of Canadian Healthy Infant Longitudinal Development (CHILD) study investigators.

Supplementary Tables

Supplementary Table 1. List of shared genera of seven included studies used in the Random Forest models to predict gut microbiota age.

Genera	Relative importance	Relative Importance (%)
k__bacteria.p__firmicutes.c__clostridia.o__clostridiales.f__lachnospiraceae.g__blautia	3264.30	27.17
k__bacteria.p__firmicutes.c__clostridia.o__clostridiales.f__lachnospiraceae.g__	1905.28	15.86
k__bacteria.p__bacteroidetes.c__bacteroidia.o__bacteroidales.f__prevotellaceae.g__prevotella	935.61	7.79
k__bacteria.p__firmicutes.c__clostridia.o__clostridiales.f.g__	903.01	7.52
k__bacteria.p__firmicutes.c__bacilli.o__bacillales.f__staphylococcaceae.g__staphylococcus	693.06	5.77
k__bacteria.p__firmicutes.c__clostridia.o__clostridiales.f__veillonellaceae.g__dialister	465.34	3.87
k__bacteria.p__firmicutes.c__bacilli.o__lactobacillales.f__lactobacillaceae.g__lactobacillus	430.94	3.59
k__bacteria.p__proteobacteria.c__gammaproteobacteria.o__pasteurellales.f__pasteurellaceae.g__haemophilus	412.80	3.44
k__bacteria.p__actinobacteria.c__actinobacteria.o__bifidobacteriales.f__bifidobacteriaceae.g__bifidobacterium	399.63	3.33
k__bacteria.p__actinobacteria.c__actinobacteria.o__actinomycetales.f__actinomycetaceae.g__actinomyces	326.51	2.72
k__bacteria.p__firmicutes.c__clostridia.o__clostridiales.f__lachnospiraceae.g__dorea	232.91	1.94
k__bacteria.p__firmicutes.c__bacilli.o__lactobacillales.f__enterococcaceae.g__enterococcus	211.03	1.76
k__bacteria.p__firmicutes.c__clostridia.o__clostridiales.f__lachnospiraceae.g__coprococcus	184.24	1.53
k__bacteria.p__firmicutes.c__bacilli.o__lactobacillales.f__streptococcaceae.g__streptococcus	180.88	1.51
k__bacteria.p__actinobacteria.c__coriobacteriia.o__coriobacteriales.f__coriobacteriaceae.g__collinsella	157.80	1.31
k__bacteria.p__firmicutes.c__clostridia.o__clostridiales.f__veillonellaceae.g__veillonella	157.13	1.31
k__bacteria.p__firmicutes.c__clostridia.o__clostridiales.f__clostridiaceae.g__clostridium	132.33	1.10
k__bacteria.p__firmicutes.c__clostridia.o__clostridiales.f__ruminococcaceae.g__oscillospira	127.32	1.06
k__bacteria.p__bacteroidetes.c__bacteroidia.o__bacteroidales.f__bacteroidaceae.g__bacteroides	125.65	1.05
k__bacteria.p__proteobacteria.c__gammaproteobacteria.o__pseudomonadales.f__pseudomonadaceae.g__pseudomonas	117.28	0.98
k__bacteria.p__actinobacteria.c__actinobacteria.o__actinomycetales.f__micrococcaceae.g__rothia	105.70	0.88
k__bacteria.p__actinobacteria.c__coriobacteriia.o__coriobacteriales.f__coriobacteriaceae.g__	100.02	0.83
k__bacteria.p__actinobacteria.c__coriobacteriia.o__coriobacteriales.f__coriobacteriaceae.g__atopobium	66.97	0.56
k__bacteria.p__proteobacteria.c__betaproteobacteria.o__neisseriales.f__neisseriaceae.g__neisseria	59.31	0.49
k__bacteria.p__bacteroidetes.c__bacteroidia.o__bacteroidales.f__porphyromonadaceae.g__parabacteroides	54.37	0.45
k__bacteria.p__proteobacteria.c__betaproteobacteria.o__burkholderiales.f__alcaligenaceae.g__sutterella	53.38	0.44
k__bacteria.p__fusobacteria.c__fusobacteriia.o__fusobacteriales.f__fusobacteriaceae.g__fusobacterium	52.32	0.44
k__bacteria.p__firmicutes.c__bacilli.o__gemellales.f__gemellaceae.g__	51.81	0.43
k__bacteria.p__firmicutes.c__bacilli.o__lactobacillales.f__streptococcaceae.g__lactococcus	44.64	0.37
k__bacteria.p__bacteroidetes.c__bacteroidia.o__bacteroidales.f__rikenellaceae.g__	32.71	0.27
k__bacteria.p__cyanobacteria.c__chloroplast.o__streptophyta.f.g__	10.73	0.09
k__bacteria.p__firmicutes.c__clostridia.o__clostridiales.f__veillonellaceae.g__acidaminococcus	7.19	0.06
k__bacteria.p__firmicutes.c__clostridia.o__clostridiales.f__lachnospiraceae.g__roseburia	6.31	0.05
k__bacteria.p__firmicutes.c__bacilli.o__lactobacillales.f__carnobacteriaceae.g__granulicatella	4.29	0.04
k__bacteria.p__firmicutes.c__bacilli.o__bacillales.f__paenibacillaceae.g__paenibacillus	0.14	0.00
k__bacteria.p__proteobacteria.c__alphaproteobacteria.o__sphingomonadales.f__sphingomonadaceae.g__sphingomonas	0.11	0.00

Full original genera names output from QIIME are shown to facilitate reproducibility.

Supplementary Table 2. Meta-analysis of all seven included studies for gut bacterial taxa with differential relative abundances between non-exclusively breastfed vs. exclusively breastfed infants ≤6 months of age.

**Bacterial taxa**				**Pooled estimate (log(OR))**	**95% Pooled lower limit**	**95% Pooled upper limit**	**Pooled p-value**	**FDR adjusted pooled p-value**
**Phylum**	**Order**	**Family**	**Genus**					
Firmicutes				0.25	0.11	0.38	3e-04	0.0018
Bacteroidetes				0.21	0.06	0.36	6e-03	0.0180
Order								
Firmicutes	Clostridiales			0.30	0.12	0.48	0.0009	0.0106
Bacteroidetes	Bacteroidales			0.21	0.06	0.36	0.0056	0.0369
Firmicutes	Erysipelotrichales			0.20	0.02	0.38	0.0253	0.1170
Family								
Firmicutes	Clostridiales	Veillonellaceae		0.23	0.06	0.40	0.0070	0.0757
Bacteroidetes	Bacteroidales	Bacteroidaceae		0.21	0.05	0.37	0.0090	0.0856
Firmicutes	Erysipelotrichales	Erysipelotrichaceae		0.20	0.02	0.38	0.0253	0.1748
Firmicutes	Clostridiales	Clostridiaceae		0.17	0.00	0.33	0.0496	0.3087
Genus								
Firmicutes	Erysipelotrichales	Erysipelotrichaceae	*.eubacterium.*	0.39	0.15	0.64	0.0015	0.0561
Firmicutes	Clostridiales	Veillonellaceae	*Megasphaera*	0.40	0.12	0.68	0.0047	0.1115
Bacteroidetes	Bacteroidales	Bacteroidaceae	*Bacteroides*	0.21	0.05	0.37	0.0090	0.1220
Firmicutes	Clostridiales	Lachnospiraceae	*Clostridium*	0.35	0.05	0.64	0.0218	0.2513
Firmicutes	Clostridiales	Veillonellaceae	*Veillonella*	0.21	0.01	0.42	0.0386	0.3598

Only those with pooled p-values <0.05 are shown. OR: odds ratio; FDR: false discovery rate.

Supplementary Table 3. Meta-analysis of five studies that included a non-breastfeeding group for gut bacterial taxa with trend in relative abundance across exclusive breastfeeding, non-exclusive breastfeeding and non-breastfeeding groups.

**Bacterial taxa**				**Pooled estimate (log(OR))**	**95% Pooled lower limit**	**95% Pooled upper limit**	**Pooled p-value**	**FDR adjusted pooled p-value**
**Phylum**	**Order**	**Family**	**Genus**					
Firmicutes				0.29	0.13	0.45	0.0004	0.0022
Verrucomicrobia				0.19	0.03	0.35	0.0172	0.0517
Bacteroidetes				0.22	0.02	0.41	0.0277	0.0553
Order								
Firmicutes	Clostridiales			0.39	0.27	0.52	<0.0001	<0.0001
Actinobacteria	Coriobacteriales			0.32	0.21	0.42	<0.0001	<0.0001
Proteobacteria	Pasteurellales			-0.22	-0.34	-0.09	0.0007	0.0045
Firmicutes	Bacillales			-0.17	-0.28	-0.07	0.0009	0.0050
Verrucomicrobia	Verrucomicrobiales			0.19	0.03	0.35	0.0172	0.0637
Bacteroidetes	Bacteroidales			0.23	0.02	0.44	0.0339	0.0965
Firmicutes	Erysipelotrichales			0.20	0.01	0.39	0.0392	0.0966
Family								
Actinobacteria	Coriobacteriales	Coriobacteriaceae		0.32	0.21	0.42	<0.0001	<0.0001
Proteobacteria	Pasteurellales	Pasteurellaceae		-0.22	-0.34	-0.09	0.0007	0.0070
Firmicutes	Bacillales	Staphylococcaceae		-0.18	-0.30	-0.07	0.0021	0.0160
Firmicutes	Clostridiales	Peptostreptococcaceae		0.19	0.05	0.33	0.0084	0.0577
Verrucomicrobia	Verrucomicrobiales	Verrucomicrobiaceae		0.19	0.03	0.35	0.0172	0.0873
Firmicutes	Clostridiales	Ruminococcaceae		0.20	0.03	0.38	0.0194	0.0923
Firmicutes	Clostridiales	Clostridiaceae		0.14	0.01	0.27	0.0334	0.1234
Firmicutes	Clostridiales	Lachnospiraceae		0.34	0.03	0.65	0.0341	0.1234
Firmicutes	Erysipelotrichales	Erysipelotrichaceae		0.20	0.01	0.39	0.0392	0.1240
Genus								
Firmicutes	Clostridiales	Lachnospiraceae	*Coprococcus*	0.33	0.32	0.34	<0.0001	<0.0001
Firmicutes	Clostridiales	Lachnospiraceae	*Blautia*	0.38	0.25	0.51	<0.0001	<0.0001
Firmicutes	Erysipelotrichales	Erysipelotrichaceae	*.eubacterium.*	0.38	0.23	0.53	<0.0001	<0.0001
Firmicutes	Clostridiales	Lachnospiraceae	*Unassigned*	0.34	0.18	0.49	<0.0001	0.0003
Proteobacteria	Pasteurellales	Pasteurellaceae	*Haemophilus*	-0.23	-0.36	-0.10	0.0005	0.0064
Firmicutes	Bacillales	Staphylococcaceae	*Staphylococcus*	-0.18	-0.30	-0.07	0.0021	0.0196
Verrucomicrobia	Verrucomicrobiales	Verrucomicrobiaceae	*Akkermansia*	0.19	0.03	0.35	0.0169	0.1167
Firmicutes	Clostridiales	Peptostreptococcaceae	*Unassigned*	0.29	0.01	0.57	0.0390	0.1823

Five studies included are Bangladesh, Canada, USA (California-Florida), USA (California-Massachusetts –Missouri), USA (North Carolina).

In each study, to test for trend across breastfeeding categories, breastfeeding was coded as a continuous variable in the model (exclusive breastfeeding (EBF)=1, non-EBF=2 and non-BF=3).

Only those with pooled p-values <0.05 are shown. OR: odds ratio; FDR: false discovery rate.

Supplementary Table 4. Meta-analysis of six studies without data from North Carolina study for gut bacterial taxa with differential relative abundances between non-exclusively breastfed vs. exclusively breastfed infants ≤6 months of age (sensitivity analysis).

**Bacterial taxa**				**Pooled estimate (log(OR))**	**95% Pooled lower limit**	**95% Pooled upper limit**	**Pooled p-value**	**FDR adjusted pooled p-value**
**Phylum**	**Order**	**Family**	**Genus**					
Firmicutes				0.25	0.11	0.38	0.0004	0.0025
Bacteroidetes				0.19	0.04	0.34	0.0114	0.0399
Order								
Firmicutes	Clostridiales			0.28	0.11	0.46	0.0015	0.0233
Bacteroidetes	Bacteroidales			0.20	0.05	0.35	0.0106	0.0873
Firmicutes	Erysipelotrichales			0.20	0.02	0.38	0.0285	0.1638
Family								
Bacteroidetes	Bacteroidales	Bacteroidaceae		0.19	0.03	0.35	0.0169	0.2092
Firmicutes	Clostridiales	Veillonellaceae		0.22	0.04	0.41	0.0176	0.2092
Firmicutes	Erysipelotrichales	Erysipelotrichaceae		0.20	0.02	0.38	0.0285	0.2460
Genus								
Firmicutes	Erysipelotrichales	Erysipelotrichaceae	*.eubacterium.*	0.40	0.15	0.65	0.0019	0.0888
Firmicutes	Clostridiales	Veillonellaceae	*Megasphaera*	0.40	0.12	0.68	0.0047	0.1804
Firmicutes	Clostridiales	Clostridiaceae	*Unassigned*	0.24	0.06	0.43	0.0101	0.2417
Bacteroidetes	Bacteroidales	Bacteroidaceae	*Bacteroides*	0.19	0.03	0.35	0.0169	0.2804
Firmicutes	Clostridiales	Lachnospiraceae	*Clostridium*	0.35	0.05	0.64	0.0218	0.3203

Only those with pooled p-values <0.05 are shown. OR: odds ratio; FDR: false discovery rate.

Supplementary Table 5. Meta-analysis of six studies without data from Haiti study for gut bacterial taxa with differential relative abundances between non-exclusively breastfed vs. exclusively breastfed infants ≤6 months of age (sensitivity analysis).

**Bacterial taxa**				**Pooled estimate (log(OR))**	**95% Pooled lower limit**	**95% Pooled upper limit**	**Pooled p-value**	**FDR adjusted pooled p-value**
**Phylum**	**Order**	**Family**	**Genus**					
Firmicutes				0.26	0.12	0.40	0.0002	0.0013
Bacteroidetes				0.21	0.06	0.36	0.0055	0.0165
Order								
Firmicutes	Clostridiales			0.32	0.18	0.46	<0.0001	0.0001
Bacteroidetes	Bacteroidales			0.22	0.07	0.37	0.0043	0.0325
Firmicutes	Erysipelotrichales			0.18	0.00	0.36	0.0444	0.2108
Family								
Bacteroidetes	Bacteroidales	Bacteroidaceae		0.21	0.04	0.37	0.0123	0.1348
Firmicutes	Clostridiales	Veillonellaceae		0.21	0.02	0.40	0.0335	0.2947
Firmicutes	Erysipelotrichales	Erysipelotrichaceae		0.18	0.00	0.36	0.0444	0.2947
Firmicutes	Clostridiales	Clostridiaceae		0.17	0.00	0.34	0.0459	0.2947
Genus								
Firmicutes	Clostridiales	Veillonellaceae	*Megasphaera*	0.45	0.15	0.75	0.0029	0.1063
Firmicutes	Erysipelotrichales	Erysipelotrichaceae	*.eubacterium.*	0.36	0.11	0.61	0.0056	0.1063
Bacteroidetes	Bacteroidales	Bacteroidaceae	*Bacteroides*	0.21	0.04	0.37	0.0123	0.1862
Firmicutes	Clostridiales	Lachnospiraceae	*Clostridium*	0.36	0.06	0.65	0.0194	0.2677
Firmicutes	Clostridiales	Lachnospiraceae	*Coprococcus*	0.52	0.05	0.99	0.0303	0.3840
Firmicutes	Clostridiales	Clostridiaceae	*Unassigned*	0.53	0.03	1.04	0.0388	0.3878

Only those with pooled p-values <0.05 are shown. OR: odds ratio; FDR: false discovery rate.

Supplementary Table 6. Meta-analysis of six studies without data from VDAART trial study for gut bacterial taxa with differential relative abundances between non-exclusively breastfed vs. exclusively breastfed infants ≤6 months of age (sensitivity analysis).

**Bacterial taxa**				**Pooled estimate (log(OR))**	**95% Pooled lower limit**	**95% Pooled upper limit**	**Pooled p-value**	**FDR adjusted pooled p-value**
**Phylum**	**Order**	**Family**	**Genus**					
Bacteroidetes				0.23	0.07	0.39	0.0043	0.0251
Firmicutes				0.20	0.05	0.35	0.0072	0.0251
Order								
Bacteroidetes	Bacteroidales			0.23	0.07	0.39	0.0040	0.0549
Firmicutes	Clostridiales			0.30	0.08	0.51	0.0069	0.0549
Actinobacteria	Actinomycetales			-0.16	-0.28	-0.03	0.0156	0.1025
Firmicutes	Erysipelotrichales			0.21	0.02	0.41	0.0331	0.1692
Firmicutes	Bacillales			-0.21	-0.41	-0.01	0.0427	0.1965
Family								
Firmicutes	Clostridiales	Veillonellaceae		0.30	0.15	0.44	0.0001	0.0050
Bacteroidetes	Bacteroidales	Bacteroidaceae		0.24	0.07	0.41	0.0064	0.0851
Firmicutes	Erysipelotrichales	Erysipelotrichaceae		0.21	0.02	0.41	0.0331	0.2621
Genus								
Firmicutes	Clostridiales	Veillonellaceae	*Acidaminococcus*	2.06	0.93	3.19	0.0003	0.0306
Firmicutes	Erysipelotrichales	Erysipelotrichaceae	*.eubacterium.*	0.41	0.14	0.68	0.0025	0.1074
Firmicutes	Clostridiales	Veillonellaceae	*Megasphaera*	0.45	0.14	0.77	0.0051	0.1074
Bacteroidetes	Bacteroidales	Bacteroidaceae.	*Bacteroides*	0.24	0.07	0.41	0.0064	0.1074
Firmicutes	Clostridiales	Veillonellaceae	*Veillonella*	0.26	0.05	0.47	0.0162	0.2080
Firmicutes	Clostridiales	Lachnospiraceae	*Clostridium*	0.35	0.05	0.64	0.0218	0.2616
Firmicutes	Clostridiales	Lachnospiraceae	*Coprococcus*	0.55	0.04	1.05	0.0334	0.3167

VDAART trial study is also referred to as USA(California-Massachusetts-Missouri) in manuscript text. Only those with pooled p-values <0.05 are shown. OR: odds ratio; FDR: false discovery rate.

Supplementary Table 7. Meta-analysis stratified by mode of delivery for gut bacterial taxa with differential relative abundances between non-exclusively breastfed vs. exclusively breastfed infants ≤6 months of age.

				Pooled estimate (log(OR))	95% Pooled lower limit	95% Pooled upper limit	Pooled p-value	FDR adjusted pooled p-value
**Vaginally born infants**								
**Phylum**	**Order**	**Family**	**Genus**					
Proteobacteria				-0.31	-0.51	-0.11	0.0025	0.0178
Order								
Proteobacteria	Enterobacteriales			-0.30	-0.51	-0.09	0.0054	0.1158
Firmicutes	Erysipelotrichales			0.28	0.04	0.52	0.0217	0.2337
Family								
Firmicutes	Bacillales	Staphylococcaceae		-0.33	-0.56	-0.10	0.0046	0.1157
Proteobacteria	Enterobacteriales	Enterobacteriace		-0.30	-0.51	-0.09	0.0054	0.1157
Bacteroidetes	Bacteroidales	Bacteroidaceae		0.30	0.08	0.52	0.0064	0.1157
Firmicutes	Clostridiales	Eubacteriaceae		0.80	0.19	1.42	0.0104	0.1577
Firmicutes	Erysipelotrichales	Erysipelotrichaceae		0.28	0.04	0.52	0.0217	0.2198
Genus								
Firmicutes	Clostridiales	Veillonellaceae	*Acidaminococcus*	3.37	2.53	4.21	<0.0001	<0.0001
Firmicutes	Bacillales	Staphylococcaceae	*Staphylococcus*	-0.33	-0.56	-0.10	0.0046	0.1176
Firmicutes	Clostridiales	Eubacteriaceae	*Eubacterium*	0.88	0.26	1.50	0.0055	0.1176
Firmicutes	Clostridiales	Lachnospiraceae	*Unassigned*	0.32	0.09	0.55	0.0063	0.1176
Bacteroidetes	Bacteroidales	Bacteroidaceae	*Bacteroides*	0.30	0.08	0.52	0.0064	0.1176
Firmicutes	Erysipelotrichales	Erysipelotrichaceae	*.eubacterium.*	0.54	0.14	0.94	0.0079	0.1327
Proteobacteria	Enterobacteriales	Enterobacteriaceae	*Unassigned*	-0.29	-0.51	-0.06	0.0127	0.1802
Firmicutes	Clostridiales	Clostridiaceae	*Unassigned*	0.30	0.06	0.55	0.0145	0.1921
Proteobacteria	Pasteurellales	Pasteurellaceae	*Aggregatibacter*	0.57	0.09	1.06	0.0209	0.2234
Firmicutes	Lactobacillales	Streptococcaceae	*Lactococcus*	0.58	0.06	1.11	0.0293	0.2849
Firmicutes	Clostridiales	Lachnospiraceae	*Blautia*	0.36	0.00	0.73	0.0490	0.4534
**C-section born infants**								
Phylum								
Proteobacteria				-0.72	-1.05	-0.38	<0.0001	0.0002
Order								
Proteobacteria	Enterobacteriales			-0.58	-1.03	-0.12	0.0127	0.1562
Family								
Proteobacteria	Enterobacteriales	Enterobacteriace		-0.58	-1.03	-0.12	0.0127	0.2596
Genus								
Proteobacteria	Enterobacteriales	Enterobacteriace	*Proteus*	-0.26	-0.26	-0.26	<0.0001	<0.0001
Firmicutes	Clostridiales	Ruminococcaceae	*Anaerotruncus*	-2.92	-4.31	-1.54	<0.0001	0.0019
Firmicutes	Clostridiales	Veillonellaceae	*Phascolarctobacterium*	-1.88	-3.56	-0.20	0.0279	0.6490
Proteobacteria	Enterobacteriales	Enterobacteriace	*Unassigned*	-0.52	-1.03	-0.01	0.0451	0.9154

Four studies included are Canada, Haiti, USA (California-Florida), USA (California-Massachusetts –Missouri).

Only those with pooled p-values <0.05 are shown. OR: odds ratio; FDR: false discovery rate.

Supplementary Table 8. Meta-analysis of all seven included studies for gut bacterial KEGG pathways with differential relative abundances between non-exclusively breastfed vs. exclusively breastfed infants ≤6 months of age.

KEGG pathway	Pooled estimate (log(OR))	95% Pooled lower limit	95% Pooled upper limit	Pooled p-value	FDR adjusted pooled p-value
Level 2 KEGG pathway					
Environmental Information Processing; Signaling Molecules and Interaction	-0.05	-0.08	-0.01	0.0056	0.1417
Genetic Information Processing; Transcription	0.02	0.00	0.03	0.0077	0.1417
Metabolism; Carbohydrate Metabolism	0.01	0.00	0.03	0.0290	0.3580
Level 3 KEGG pathway					
Metabolism; Carbohydrate Metabolism; Fructose and mannose metabolism	0.08	0.05	0.11	<0.0001	0.0001
Cellular Processes; Transport and Catabolism; Peroxisome	-0.06	-0.09	-0.03	<0.0001	0.0021
Metabolism; Lipid Metabolism; Fatty acid metabolism	-0.09	-0.14	-0.04	0.0002	0.0180
Metabolism; Carbohydrate Metabolism; Pentose and glucuronate interconversions	0.06	0.03	0.10	0.0007	0.0380
Metabolism; Lipid Metabolism; Fatty acid biosynthesis	0.04	0.02	0.07	0.0009	0.0380
Metabolism; Metabolism of Cofactors and Vitamins; Vitamin B6 metabolism	-0.03	-0.05	-0.01	0.0011	0.0390
Metabolism; Metabolism of Terpenoids and Polyketides; Biosynthesis of ansamycins	0.08	0.03	0.13	0.0014	0.0441
Metabolism; Carbohydrate Metabolism; Pentose phosphate pathway	0.04	0.01	0.06	0.0021	0.0595
Genetic Information Processing; Translation; Ribosome biogenesis in eukaryotes	-0.07	-0.12	-0.02	0.0041	0.1012
Organismal Systems; Endocrine System; Adipocytokine signaling pathway	-0.12	-0.20	-0.04	0.0048	0.1080
Genetic Information Processing; Replication and Repair; Base excision repair	0.01	0.00	0.03	0.0058	0.1178
Metabolism; Xenobiotics Biodegradation and Metabolism; Xylene degradation	-0.04	-0.08	-0.01	0.0125	0.2194
Metabolism; Energy Metabolism; Carbon fixation in photosynthetic organisms	0.03	0.01	0.06	0.0129	0.2194
Metabolism; Xenobiotics Biodegradation and Metabolism; Drug metabolism (other enzymes)	0.03	0.01	0.06	0.0138	0.2194
Unclassified; Genetic Information Processing; Protein folding and associated processing	-0.01	-0.02	0.00	0.0210	0.3120
Metabolism; Metabolism of Cofactors and Vitamins; Nicotinate and nicotinamide metabolism	-0.03	-0.06	0.00	0.0265	0.3293
Unclassified; Cellular Processes and Signaling; Inorganic ion transport and metabolism	-0.07	-0.14	-0.01	0.0270	0.3293
Organismal Systems; Endocrine System; Insulin signaling pathway	0.06	0.01	0.11	0.0286	0.3293
Metabolism; Carbohydrate Metabolism; Amino sugar and nucleotide sugar metabolism	0.03	0.00	0.06	0.0316	0.3293
Metabolism; Metabolism of Other Amino Acids; D Alanine metabolism	0.02	0.00	0.04	0.0320	0.3293
Metabolism; Xenobiotics Biodegradation and Metabolism; Drug metabolism (cytochrome P450)	-0.10	-0.19	-0.01	0.0326	0.3293
Metabolism; Xenobiotics Biodegradation and Metabolism; Toluene degradation	-0.07	-0.14	-0.01	0.0337	0.3293
Metabolism; Lipid Metabolism; Glycerolipid metabolism	0.03	0.00	0.07	0.0340	0.3293
Metabolism; Glycan Biosynthesis and Metabolism; Glycosphingolipid biosynthesis (globo series)	0.08	0.00	0.16	0.0459	0.4265

Only those with pooled p-values <0.05 are shown. KEGG: Kyoto Encyclopedia of Genes and Genomes; OR: odds ratio; FDR: false discovery rate.

Supplementary Table 9. Meta-analysis of included studies without data from either North Carolina, Haiti or VDAART trial study for gut bacterial KEGG pathways at level 3 with differential relative abundances between non-exclusively breastfed vs. exclusively breastfed infants ≤6 months of age (sensitivity analysis).

Level 3 KEGG pathways	Pooled estimate (log(OR))	95% Pooled lower limit	95% Pooled upper limit	Pooled p-value	FDR adjusted pooled p-value
**Meta-analysis without North Carolina data**					
Metabolism; Carbohydrate Metabolism; Fructose and mannose metabolism	0.07	0.04	0.10	<0.0001	0.0002
Cellular Processes; Transport and Catabolism; Peroxisome	-0.06	-0.09	-0.03	<0.0001	0.0026
Metabolism; Lipid Metabolism; Fatty acid metabolism	-0.09	-0.14	-0.04	0.0008	0.0596
Genetic Information Processing; Replication and Repair; Base excision repair	0.02	0.01	0.03	0.0019	0.0875
Metabolism; Carbohydrate Metabolism; Pentose and glucuronate interconversions	0.06	0.02	0.10	0.0021	0.0875
Metabolism; Metabolism of Terpenoids and Polyketides; Biosynthesis of ansamycins	0.08	0.03	0.12	0.0023	0.0875
Metabolism; Metabolism of Cofactors and Vitamins; Vitamin B6 metabolism	-0.03	-0.05	-0.01	0.0030	0.0954
Metabolism; Lipid Metabolism; Fatty acid biosynthesis	0.04	0.01	0.07	0.0038	0.1005
Genetic Information Processing; Translation; Ribosome biogenesis in eukaryotes	-0.07	-0.12	-0.02	0.0040	0.1005
Metabolism; Carbohydrate Metabolism; Pentose phosphate pathway	0.03	0.01	0.06	0.0057	0.1298
Metabolism; Metabolism of Other Amino Acids; D Alanine metabolism	0.02	0.00	0.04	0.0227	0.4660
Unclassified; Genetic Information Processing; Protein folding and associated processing	-0.01	-0.02	0.00	0.0270	0.5078
Metabolism; Xenobiotics Biodegradation and Metabolism; Drug metabolism…other enzymes	0.03	0.00	0.06	0.0321	0.5078
Organismal Systems; Endocrine System; Adipocytokine signaling pathway	-0.10	-0.20	-0.01	0.0351	0.5078
Metabolism; Energy Metabolism; Carbon fixation in photosynthetic organisms	0.03	0.00	0.05	0.0357	0.5078
Metabolism; Xenobiotics Biodegradation and Metabolism; Drug metabolism (cytochrome P450)	-0.10	-0.19	-0.01	0.0359	0.5078
Human Diseases; Infectious Diseases; Epithelial cell signaling in Helicobacter pylori infection	0.05	0.00	0.09	0.0414	0.5378
Metabolism; Metabolism of Cofactors and Vitamins; Nicotinate and nicotinamide metabolism	-0.03	-0.06	0.00	0.0447	0.5378
Metabolism; Carbohydrate Metabolism; Amino sugar and nucleotide sugar metabolism	0.03	0.00	0.06	0.0488	0.5378
**Meta-analysis without Haiti data**					
Metabolism; Carbohydrate Metabolism; Fructose and mannose metabolism	0.08	0.05	0.11	<0.0001	0.0002
Cellular Processes; Transport and Catabolism; Peroxisome	-0.07	-0.09	-0.04	<0.0001	0.0016
Metabolism; Lipid Metabolism; Fatty acid metabolism	-0.09	-0.14	-0.05	<0.0001	0.0031
Metabolism; Carbohydrate Metabolism; Pentose and glucuronate interconversions	0.07	0.03	0.10	0.0003	0.0161
Metabolism; Metabolism of Terpenoids and Polyketides; Biosynthesis of ansamycins	0.09	0.04	0.14	0.0004	0.0184
Metabolism; Carbohydrate Metabolism; Pentose phosphate pathway	0.04	0.02	0.06	0.0009	0.0302
Organismal Systems; Environmental Adaptation; Plant pathogen interaction	0.05	0.02	0.07	0.0009	0.0302
Metabolism; Lipid Metabolism; Fatty acid biosynthesis	0.04	0.02	0.07	0.0019	0.0528
Organismal Systems; Endocrine System; Adipocytokine signaling pathway	-0.13	-0.21	-0.05	0.0025	0.0617
Genetic Information Processing; Translation; Ribosome biogenesis in eukaryotes	-0.07	-0.12	-0.02	0.0034	0.0767
Metabolism; Metabolism of Cofactors and Vitamins; Vitamin B6 metabolism	-0.03	-0.05	-0.01	0.0039	0.0791
Metabolism; Metabolism of Cofactors and Vitamins; Porphyrin and chlorophyll metabolism	0.06	0.02	0.11	0.0051	0.0960
Metabolism; Energy Metabolism; Carbon fixation in photosynthetic organisms	0.04	0.01	0.06	0.0057	0.0975
Metabolism; Xenobiotics Biodegradation and Metabolism; Drug metabolism…other enzymes	0.04	0.01	0.06	0.0074	0.1192
Genetic Information Processing; Replication and Repair; Base excision repair	0.01	0.00	0.03	0.0099	0.1481
Metabolism; Metabolism of Cofactors and Vitamins; Biotin metabolism	0.04	0.01	0.08	0.0140	0.1955
Unclassified; Cellular Processes and Signaling; Inorganic ion transport and metabolism	-0.09	-0.16	-0.01	0.0188	0.2441
Metabolism; Lipid Metabolism; Glycerolipid metabolism	0.04	0.01	0.07	0.0196	0.2441
Metabolism; Xenobiotics Biodegradation and Metabolism; Toluene degradation	-0.08	-0.15	-0.01	0.0227	0.2625
Metabolism; Xenobiotics Biodegradation and Metabolism; Drug metabolism…cytochrome P450	-0.11	-0.20	-0.01	0.0234	0.2625
Organismal Systems; Endocrine System; Insulin signaling pathway	0.06	0.01	0.11	0.0281	0.2935
Unclassified; Genetic Information Processing; Protein folding and associated processing	-0.01	-0.02	0.00	0.0288	0.2935
Metabolism; Metabolism of Other Amino Acids; D Alanine metabolism	0.02	0.00	0.04	0.0329	0.3200
Metabolism; Amino Acid Metabolism; Glycine; serine and threonine metabolism	-0.02	-0.03	0.00	0.0357	0.3249
Metabolism; Metabolism of Cofactors and Vitamins; Nicotinate and nicotinamide metabolism	-0.03	-0.06	0.00	0.0369	0.3249
Metabolism; Xenobiotics Biodegradation and Metabolism; Metabolism of xenobiotics by cytochrome P450	-0.10	-0.19	-0.01	0.0387	0.3249
Genetic Information Processing; Translation; RNA transport	0.06	0.00	0.12	0.0392	0.3249
Metabolism; Metabolism of Terpenoids and Polyketides; Tetracycline biosynthesis	0.06	0.00	0.11	0.0460	0.3515
Genetic Information Processing; Folding; Sorting and Degradation; Proteasome	-0.10	-0.19	0.00	0.0466	0.3515
Human Diseases; Infectious Diseases; Epithelial cell signaling in Helicobacter pylori infection	0.05	0.00	0.09	0.0475	0.3515
Metabolism; Carbohydrate Metabolism; Amino sugar and nucleotide sugar metabolism	0.03	0.00	0.06	0.0486	0.3515
**Meta-analysis without VDAART trial data**					
Metabolism; Carbohydrate Metabolism; Fructose and mannose metabolism	0.07	0.04	0.10	<0.0001	0.0002
Cellular Processes; Transport and Catabolism; Peroxisome	-0.06	-0.09	-0.03	0.0002	0.0210
Metabolism; Xenobiotics Biodegradation and Metabolism; Xylene degradation	-0.06	-0.09	-0.02	0.0026	0.1577
Metabolism; Lipid Metabolism; Fatty acid metabolism	-0.09	-0.15	-0.03	0.0028	0.1577
Genetic Information Processing; Folding; Sorting and Degradation; Chaperones and folding catalysts	0.02	0.01	0.04	0.0036	0.1577
Genetic Information Processing; Translation; Ribosome biogenesis in eukaryotes	-0.07	-0.12	-0.02	0.0043	0.1577
Metabolism; Carbohydrate Metabolism; Pentose and glucuronate interconversions	0.05	0.02	0.09	0.0051	0.1577
Metabolism; Lipid Metabolism; Fatty acid biosynthesis	0.05	0.01	0.08	0.0056	0.1577
Metabolism; Metabolism of Terpenoids and Polyketides; Biosynthesis of ansamycins	0.07	0.02	0.13	0.0090	0.2250
Metabolism; Metabolism of Cofactors and Vitamins; Vitamin B6 metabolism	-0.02	-0.04	-0.01	0.0104	0.2302
Metabolism; Metabolism of Cofactors and Vitamins; Biotin metabolism	0.05	0.01	0.09	0.0118	0.2302

Level 3 KEGG pathways	Pooled estimate (log(OR))	95% Pooled lower limit	95% Pooled upper limit	Pooled p-value	FDR adjusted pooled p-value
Metabolism; Carbohydrate Metabolism; Pentose phosphate pathway	0.03	0.01	0.06	0.0122	0.2302
Metabolism; Xenobiotics Biodegradation and Metabolism; Drug metabolism…other enzymes	0.04	0.01	0.07	0.0171	0.2974
Metabolism; Xenobiotics Biodegradation and Metabolism; Chloroalkane and chloroalkene degradation	-0.04	-0.08	-0.01	0.0187	0.3025
Genetic Information Processing; Replication and Repair; Base excision repair	0.01	0.00	0.03	0.0214	0.3222
Metabolism; Xenobiotics Biodegradation and Metabolism; Drug metabolism…cytochrome P450	-0.10	-0.20	-0.01	0.0313	0.4117
Environmental Information Processing; Signal Transduction; Phosphatidylinositol signaling system	0.02	0.00	0.03	0.0314	0.4117
Metabolism; Energy Metabolism; Carbon fixation in photosynthetic organisms	0.03	0.00	0.07	0.0328	0.4117
Metabolism; Metabolism of Other Amino Acids; D Alanine metabolism	0.02	0.00	0.04	0.0363	0.4323
Genetic Information Processing; Folding; Sorting and Degradation; Proteasome	-0.10	-0.20	0.00	0.0427	0.4829

VDAART trial study is also referred to as USA(California-Massachusetts-Missouri) in manuscript text. Only those with pooled p-values <0.05 are shown. KEGG: Kyoto Encyclopedia of Genes and Genomes; OR: odds ratio; FDR: false discovery rate.

Supplementary Table 10. Meta-analysis stratified by mode of delivery for gut bacterial KEGG pathways with differential relative abundances between non-exclusively breastfed vs. exclusively breastfed infants ≤6 months of age.

	Pooled estimate (log(OR))	95% Pooled lower limit	95% Pooled upper limit	Pooled p-value	FDR adjusted pooled p-value
**Vaginal born infants**					
Level 2 KEGG pathways					
Human Diseases; Infectious Diseases	-0.05	-0.08	-0.02	0.0005	0.0189
Human Diseases; Neurodegenerative Diseases	-0.08	-0.14	-0.03	0.0033	0.0631
Unclassified; Genetic Information Processing	-0.02	-0.04	0.00	0.0152	0.1607
Environmental Information Processing; Signal Transduction	-0.06	-0.10	-0.01	0.0169	0.1607
Metabolism; Metabolism of Other Amino Acids	-0.02	-0.03	0.00	0.0280	0.2126
Level 3 KEGG pathways					
Metabolism; Carbohydrate Metabolism; Pentose phosphate pathway	0.05	0.03	0.06	<0.0001	0.0001
Metabolism; Carbohydrate Metabolism; Propanoate metabolism	-0.06	-0.08	-0.03	<0.0001	0.0026
Metabolism; Lipid Metabolism; Fatty acid metabolism	-0.10	-0.16	-0.04	0.0005	0.0371
Metabolism; Carbohydrate Metabolism; Fructose and mannose metabolism	0.07	0.03	0.12	0.0010	0.0593
Unclassified; Cellular Processes and Signaling; Sporulation	0.27	0.10	0.44	0.0022	0.0994
Metabolism; Carbohydrate Metabolism; Amino sugar and nucleotide sugar metabolism	0.04	0.01	0.07	0.0026	0.0994
Metabolism; Enzyme Families; Peptidases	0.02	0.01	0.04	0.0032	0.1053
Metabolism; Metabolism of Cofactors and Vitamins; Pantothenate and CoA biosynthesis	0.02	0.01	0.04	0.0079	0.2155
Metabolism; Energy Metabolism; Carbon fixation in photosynthetic organisms	0.03	0.01	0.06	0.0093	0.2155
Metabolism; Carbohydrate Metabolism; Butanoate metabolism	-0.04	-0.07	-0.01	0.0106	0.2155
Metabolism; Metabolism of Other Amino Acids; Glutathione metabolism	-0.08	-0.13	-0.02	0.0111	0.2155
Metabolism; Amino Acid Metabolism; Tryptophan metabolism	-0.10	-0.18	-0.02	0.0116	0.2155
Metabolism; Amino Acid Metabolism; Lysine degradation	-0.09	-0.17	-0.02	0.0130	0.2155
Environmental Information Processing; Membrane Transport; Bacterial secretion system	-0.05	-0.09	-0.01	0.0132	0.2155
Unclassified; Genetic Information Processing; Replication; recombination and repair proteins	-0.04	-0.08	-0.01	0.0144	0.2198
Environmental Information Processing; Signal Transduction; Two component system	-0.06	-0.11	-0.01	0.0203	0.2911
Metabolism; Carbohydrate Metabolism; Pentose and glucuronate interconversions	0.06	0.01	0.11	0.0249	0.3300
Metabolism; Lipid Metabolism; Sphingolipid metabolism	0.10	0.01	0.18	0.0259	0.3300
Metabolism; Metabolism of Terpenoids and Polyketides; Limonene and pinene degradation	-0.08	-0.16	-0.01	0.0353	0.4255
Metabolism; Amino Acid Metabolism; Valine; leucine and isoleucine degradation	-0.09	-0.17	0.00	0.0394	0.4327
Metabolism; Energy Metabolism; Methane metabolism	0.04	0.00	0.07	0.0403	0.4327
Metabolism; Metabolism of Cofactors and Vitamins; Vitamin B6 metabolism	-0.03	-0.06	0.00	0.0428	0.4327
Organismal Systems; Endocrine System; Insulin signaling pathway	0.07	0.00	0.14	0.0453	0.4327
Environmental Information Processing; Membrane Transport; Secretion system	-0.05	-0.10	0.00	0.0463	0.4327
Metabolism; Metabolism of Terpenoids and Polyketides; Biosynthesis of ansamycins	0.08	0.00	0.16	0.0472	0.4327
**C-section born infants**					
Level 2 KEGG pathways					
Human Diseases; Infectious Diseases	-0.11	-0.16	-0.06	<0.0001	0.0005
Environmental Information Processing; Signal Transduction	-0.14	-0.21	-0.07	0.0001	0.0028
Unclassified; Poorly Characterized	-0.18	-0.32	-0.04	0.0092	0.0868
Metabolism; Energy Metabolism	0.04	0.01	0.07	0.0110	0.0868
Human Diseases; Neurodegenerative Diseases	-0.14	-0.25	-0.03	0.0114	0.0868
Genetic Information Processing; Replication and Repair	0.11	0.01	0.20	0.0258	0.1311
Metabolism; Nucleotide Metabolism	0.11	0.01	0.21	0.0258	0.1311
Metabolism; Amino Acid Metabolism	0.03	0.00	0.05	0.0297	0.1311
Unclassified; Cellular Processes and Signaling	-0.15	-0.29	-0.01	0.0335	0.1311
Metabolism; Carbohydrate Metabolism	0.03	0.00	0.05	0.0345	0.1311
Level 3 KEGG pathways					
Metabolism; Amino Acid Metabolism; Lysine degradation	-0.24	-0.36	-0.13	<0.0001	0.0052
Environmental Information Processing; Membrane Transport; Secretion system	-0.17	-0.25	-0.09	<0.0001	0.0052
Cellular Processes; Cell Motility; Cytoskeleton proteins	0.19	0.10	0.29	0.0001	0.0080
Environmental Information Processing; Signal Transduction; Two component system	-0.16	-0.24	-0.07	0.0002	0.0103
Metabolism; Amino Acid Metabolism; Lysine biosynthesis	0.09	0.04	0.14	0.0004	0.0148
Metabolism; Amino Acid Metabolism; Tryptophan metabolism	-0.22	-0.34	-0.10	0.0004	0.0148
Metabolism; Metabolism of Cofactors and Vitamins; Thiamine metabolism	0.08	0.03	0.12	0.0006	0.0201
Metabolism; Lipid Metabolism; Biosynthesis of unsaturated fatty acids	-0.16	-0.25	-0.07	0.0009	0.0249
Metabolism; Xenobiotics Biodegradation and Metabolism; Drug metabolism (other enzymes)	0.07	0.03	0.11	0.0010	0.0253
Metabolism; Lipid Metabolism; Fatty acid metabolism	-0.15	-0.24	-0.06	0.0012	0.0267
Genetic Information Processing; Folding; Sorting and Degradation; Sulfur relay system	-0.14	-0.22	-0.05	0.0015	0.0300
Metabolism; Carbohydrate Metabolism; Galactose metabolism	0.12	0.04	0.19	0.0016	0.0300
Genetic Information Processing; Replication and Repair; Mismatch repair	0.08	0.03	0.14	0.0020	0.0348
Metabolism; Enzyme Families; Peptidases	0.04	0.01	0.06	0.0022	0.0362
Unclassified; Cellular Processes and Signaling; Inorganic ion transport and metabolism	-0.23	-0.38	-0.08	0.0025	0.0379
Metabolism; Biosynthesis of Other Secondary Metabolites; Phenylpropanoid biosynthesis	0.16	0.06	0.27	0.0028	0.0379
Genetic Information Processing; Replication and Repair; Nucleotide excision repair	0.19	0.07	0.31	0.0028	0.0379
Metabolism; Carbohydrate Metabolism; Amino sugar and nucleotide sugar metabolism	0.09	0.03	0.14	0.0031	0.0379
Genetic Information Processing; Replication and Repair; DNA replication proteins	0.07	0.02	0.12	0.0031	0.0379
Unclassified; Cellular Processes and Signaling; Sporulation	0.43	0.13	0.72	0.0048	0.0548
Unclassified; Cellular Processes and Signaling; Other ion coupled transporters	-0.11	-0.18	-0.03	0.0055	0.0596
Metabolism; Amino Acid Metabolism; Valine; leucine and isoleucine degradation	-0.15	-0.25	-0.04	0.0067	0.0667
Metabolism; Metabolism of Other Amino Acids; Glutathione metabolism	-0.12	-0.21	-0.03	0.0067	0.0667
Metabolism; Energy Metabolism; Carbon fixation in photosynthetic organisms	0.05	0.01	0.09	0.0070	0.0667
Unclassified; Metabolism; Metabolism of cofactors and vitamins	-0.13	-0.23	-0.04	0.0075	0.0682
Human Diseases; Infectious Diseases; Vibrio cholerae pathogenic cycle	-0.13	-0.22	-0.03	0.0079	0.0692
Metabolism; Nucleotide Metabolism; Pyrimidine metabolism	0.11	0.03	0.19	0.0101	0.0816
Metabolism; Amino Acid Metabolism; Phenylalanine; tyrosine and tryptophan biosynthesis	0.08	0.02	0.13	0.0109	0.0816
Unclassified; Poorly Characterized; Function unknown	-0.16	-0.28	-0.04	0.0110	0.0816
Unclassified; Metabolism; Biosynthesis and biodegradation of secondary metabolites	-0.22	-0.38	-0.05	0.0111	0.0816
Metabolism; Enzyme Families; Protein kinases	-0.13	-0.23	-0.03	0.0113	0.0816

Metabolism; Metabolism of Cofactors and Vitamins; One carbon pool by folate	0.14	0.03	0.26	0.0114	0.0816
Metabolism; Energy Metabolism; Methane metabolism	0.06	0.01	0.11	0.0118	0.0822
Cellular Processes; Cell Growth and Death; Cell cycle (Caulobacter)	0.21	0.04	0.37	0.0138	0.0929
Genetic Information Processing; Replication and Repair; DNA replication	0.10	0.02	0.17	0.0145	0.0950
Metabolism; Metabolism of Cofactors and Vitamins; Ubiquinone and other terpenoid quinone biosynthesis	-0.13	-0.24	-0.02	0.0168	0.1066
Human Diseases; Infectious Diseases; Pertussis	-0.35	-0.63	-0.06	0.0181	0.1119
Metabolism; Carbohydrate Metabolism; Starch and sucrose metabolism	0.10	0.02	0.18	0.0200	0.1187
Metabolism; Xenobiotics Biodegradation and Metabolism; Polycyclic aromatic hydrocarbon degradation	0.17	0.03	0.32	0.0204	0.1187
Metabolism; Metabolism of Cofactors and Vitamins; Pantothenate and CoA biosynthesis	0.04	0.01	0.07	0.0207	0.1187
Unclassified; Cellular Processes and Signaling; Electron transfer carriers	-0.32	-0.58	-0.05	0.0215	0.1199
Cellular Processes; Cell Motility; Bacterial motility proteins	-0.20	-0.37	-0.03	0.0237	0.1293
Metabolism; Amino Acid Metabolism; Amino acid related enzymes	0.10	0.01	0.19	0.0251	0.1335
Metabolism; Glycan Biosynthesis and Metabolism; Peptidoglycan biosynthesis	0.17	0.02	0.31	0.0257	0.1337
Metabolism; Glycan Biosynthesis and Metabolism; Lipopolysaccharide biosynthesis proteins	-0.49	-0.92	-0.06	0.0263	0.1337
Unclassified; Cellular Processes and Signaling; Membrane and intracellular structural molecules	-0.32	-0.61	-0.04	0.0272	0.1338
Metabolism; Energy Metabolism; Photosynthesis	0.31	0.03	0.58	0.0275	0.1338
Metabolism; Energy Metabolism; Photosynthesis proteins	0.29	0.02	0.56	0.0325	0.1420
Genetic Information Processing; Replication and Repair; Homologous recombination	0.15	0.01	0.29	0.0326	0.1420
Genetic Information Processing; Folding; Sorting and Degradation; Protein export	0.20	0.02	0.38	0.0327	0.1420
Metabolism; Lipid Metabolism; Sphingolipid metabolism	0.32	0.03	0.61	0.0332	0.1420
Genetic Information Processing; Translation; Translation factors	0.20	0.02	0.38	0.0338	0.1420
Genetic Information Processing; Replication and Repair; DNA repair and recombination proteins	0.14	0.01	0.27	0.0342	0.1420
Metabolism; Metabolism of Terpenoids and Polyketides; Prenyltransferases	0.07	0.01	0.13	0.0345	0.1420
Unclassified; Poorly Characterized; General function prediction only	-0.15	-0.29	-0.01	0.0345	0.1420
Unclassified; Cellular Processes and Signaling; Signal transduction mechanisms	-0.12	-0.23	-0.01	0.0347	0.1420
Metabolism; Metabolism of Other Amino Acids; beta Alanine metabolism	-0.15	-0.29	-0.01	0.0409	0.1643
Metabolism; Metabolism of Terpenoids and Polyketides; Limonene and pinene degradation	-0.18	-0.36	-0.01	0.0418	0.1652
Metabolism; Metabolism of Terpenoids and Polyketides; Biosynthesis of siderophore group nonribosomal peptides	-0.28	-0.55	-0.01	0.0437	0.1690
Genetic Information Processing; Translation; Ribosome	0.26	0.01	0.52	0.0443	0.1690
Metabolism; Xenobiotics Biodegradation and Metabolism; Toluene degradation	-0.09	-0.19	0.00	0.0457	0.1717
Genetic Information Processing; Replication and Repair; Chromosome	0.03	0.00	0.06	0.0467	0.1725
Metabolism; Carbohydrate Metabolism; Glyoxylate and dicarboxylate metabolism	-0.10	-0.19	0.00	0.0486	0.1768

Four studies included are Canada, Haiti, USA (California-Florida), the VDAART trial or USA (California-Massachusetts –Missouri).

Only those with pooled p-values <0.05 are shown. KEGG: Kyoto Encyclopedia of Genes and Genomes; OR: odds ratio; FDR: false discovery rate.

Supplementary Table 11. Gut bacterial taxa with differential relative abundances from 6 months to 2 years of age between infants with duration of exclusive breastfeeding >2 months vs. ≤2 months from birth.

**Bacterial taxa**				**Estimate (log(OR))**	**95% Lower limit**	**95% Upper limit**	**p-value**	**FDR adjusted p-value**
**Phylum**	**Order**	**Family**	**Genus**					
Firmicutes				-0.25	-0.37	-0.12	0.0001	0.0005
Actinobacteria				0.23	0.09	0.37	0.0015	0.0029
Order								
Actinobacteria	Coriobacteriales			-0.25	-0.38	-0.12	0.0002	0.0007
Firmicutes	Lactobacillales			-0.27	-0.41	-0.13	0.0002	0.0007
Actinobacteria	Bifidobacteriales			0.25	0.11	0.39	0.0004	0.0009
Firmicutes	Erysipelotrichales			-0.15	-0.30	0.00	0.0448	0.0784
Family								
Firmicutes	Lactobacillales	Lactobacillaceae		-0.31	-0.46	-0.16	<0.0001	0.0006
Actinobacteria	Coriobacteriales	Coriobacteriaceae		-0.25	-0.38	-0.12	0.0002	0.0001
Actinobacteria	Bifidobacteriales	Bifidobacteriaceae		0.25	0.11	0.39	0.0004	0.0017
Bacteroidetes	Bacteroidales	Prevotellaceae		-0.27	-0.43	-0.11	0.0008	0.0027
Firmicutes	Clostridiales	Clostridiaceae		-0.22	-0.37	-0.06	0.0067	0.0174
Firmicutes	Lactobacillales	Enterococcaceae		0.19	0.03	0.35	0.0193	0.0419
Firmicutes	Erysipelotrichales	Erysipelotrichaceae		-0.15	-0.30	0.00	0.0448	0.0789
Firmicutes	Clostridiales	Lachnospiraceae		-0.14	-0.28	0.00	0.0486	0.0789
Genus								
Firmicutes	Lactobacillales	Lactobacillaceae	*Lactobacillus*	-0.33	-0.48	-0.18	<0.0001	0.0003
Firmicutes	Clostridiales	Unassigned	*Unassigned*	-0.34	-0.49	-0.18	<0.0001	0.0003
Actinobacteria	Bifidobacteriales	Bifidobacteriaceae	*Bifidobacterium*	0.25	0.11	0.39	0.0004	0.0027
Firmicutes	Clostridiales	Lachnospiraceae	*.ruminococcus.*	-0.25	-0.40	-0.11	0.0007	0.0035
Bacteroidetes	Bacteroidales	Prevotellaceae	*Prevotella*	-0.27	-0.43	-0.11	0.0008	0.0035
Actinobacteria	Coriobacteriales	Coriobacteriaceae	*Unassigned*	-0.22	-0.36	-0.08	0.0027	0.0093
Firmicutes	Clostridiales	Clostridiaceae	*Unassigned*	-0.24	-0.41	-0.08	0.0043	0.0129
Firmicutes	Clostridiales	Lachnospiraceae	*Blautia*	-0.22	-0.37	-0.06	0.0058	0.0152
Actinobacteria	Coriobacteriales	Coriobacteriaceae	*Collinsella*	-0.17	-0.30	-0.04	0.0125	0.0292
Firmicutes	Erysipelotrichales	Erysipelotrichaceae	*Catenibacterium*	-0.23	-0.42	-0.04	0.0165	0.0346
Firmicutes	Clostridiales	Lachnospiraceae	*Unassigned*	-0.16	-0.30	-0.02	0.0259	0.0494
Firmicutes	Lactobacillales	Enterococcaceae	*Enterococcus*	0.18	0.02	0.34	0.0310	0.0543

Data from Bangladesh study only.

Only those with p-values <0.05 are shown. OR: odds ratio; FDR: false discovery rate.

Supplementary Table 12. Breastfeeding is associated with reduced differences in gut bacterial taxa relative abundances between those who had vs. did not have diarrhea at the time of stool sample collection in infants from 6 months to 2 years of age.

	Estimate (log(OR))	95% Lower limit	95% Upper limit	p-value	FDR adjusted p-value
**Stratified by duration of exclusive breastfeeding (EBF)**					
In infants with duration of EBF <=2 months (diarrhea vs. no diarrhea comparison)					
Bifidobacteriaceae	-0.77	-1.16	-0.38	0.0001	0.0017
Coriobacteriaceae	-0.69	-1.08	-0.31	0.0005	0.0031
Streptococcaceae	0.53	0.17	0.89	0.0044	0.0191
In infants with duration of EBF >2 months (diarrhea vs. no diarrhea comparison)					
No bacterial family with change in relative abundance p-value <0.05					
**Stratified by breastfeeding status at the time of diarrhea**					
In infants without breastfeeding when diarrhea (diarrhea vs. no diarrhea comparison)					
Streptococcaceae	2.09	0.87	3.32	0.0018	0.0228
Bifidobacteriaceae	-1.84	-3.34	-0.34	0.0208	0.1351
In infants with breastfeeding when diarrhea (diarrhea vs. no diarrhea comparison)					
Coriobacteriaceae	-0.34	-0.63	-0.06	0.0189	0.2458
Bifidobacteriaceae	-0.25	-0.55	0.05	0.0981	0.4727

Data from Bangladesh study only.

Only taxa with p-value <0.05 are shown. OR: odds ratio; FDR: false discovery rate.

Supplementary Table 13. Additional summary of included studies.

Data origin (study population), reference	Results about the effects of breastfeeding on infant gut microbiome and statistical methods used in published paper	Starting files used and data processing done in this project	Note
Bangladesh (Subramanian et al. 2014) [34] #€*	No analysis on breastfeeding status	Assembled 16S reads used for OTU picking (.fna file), mapping and meta-data files.<br>- Open OTU picking with UCLUST with 97% similarity using the Greengenes database (version 13.8)	Total number of all samples from birth to 2 years of age =996 (EBF=152, non-EBF=794, non-BF=50)
Canada (Azad et al. 2015) [30] #$	- diversity (Chao1, Shannon): trend test significant (EBF<Non-EBF<non-BF)<br>- composition: (Kruskal–Wallis test):<br>EBF>Non-EBF>non-BF: Proteobacteria, Actinobacteria;<br>EBF<Non-EBF<non-BF: Bacteroidetes, Firmicutes (Clostridiales)	- Bacterial taxa relative abundance summary tables from phylum to genus levels; alpha diversity summary tables; predicted KEGG pathway abundance summary tables; metadata file. (Prior sequence data processing was done by the collaborative group using similar procedures as of this project).	The effect of infant age on breastfeeding status and gut microbiome was not accounted in the analysis (relatively accounted by study design (stool sample collection at similar age))
Haiti (Bender et al. 2016) [3] $€	PERMANNOVA test: no significant difference between EBF and non-EBF	- Assembled 16S reads used for OUT picking (.fna file), mapping and meta-data files.<br>- Open reference OTU picking with UCLUST with 97% similarity using the Greengenes database (version 13.8)	The effect of infant age on breastfeeding status and gut microbiome was not accounted in the analysis (relatively accounted by study design(stool sample collection at similar age))
South Africa (Wood et al. 2018) [27]	- Alpha diversity (Chao1): non-EBF >EBF (Wilcoxon's test)<br>- Bacterial composition (DESeq2 package (negative binomial generalized linear models)):<br>- EBF > non-EBF: *S. lactarius*, *Actinomyces*, *Atopobium*<br>- non-EBF > EBF: *Streptococcus. luteciae*, Bacteroides (OTU)	- Assembled raw FASTQ file.<br>- Converting FASTQ file to FASTA and QUAL files. Open reference OTU picking with UCLUST with 97% similarity using the Greengenes database (version 13.8)	The effect of infant age on breastfeeding status and gut microbiome was accounted by study design (stool sample collection at the same age)
USA (California and Florida) (Pannaraj et al. 2017) [5] #$€	Bacterial composition (Random Forest model):<br>- EBF > non-EBF: Proteobacteria, Bacteroides.<br>- non-EBF >EBF: Firmicutes, Actinobacteria	- Assembled 16S reads used for OTU picking (.fna file), mapping and meta-data files.<br>- Open reference OTU picking with UCLUST with 97% similarity using the Greengenes database (version 13.8)	The effect of infant age on breastfeeding status and gut microbiome was not accounted in the analysis
USA (Massachusetts, Missouri, and California)<br>(Sordillo et al. 2017) [18] #$	No direct comparison between EBF and non-EBF (EBF and non-EBF was compared with formula fed)	- Bacterial taxa relative abundance summary tables from phylum to genus levels; alpha diversity summary tables of different rarefaction depth; predicted KEGG pathway abundance summary tables; metadata file. (Prior sequence data processing was done by the collaborative group using similar procedures as of this project).	The effect of infant age on gut microbiome was accounted in the analysis
USA (North Carolina) (Thompson et al. 2015) [28] #€	(non-parametric test ANOSIM (Analysis of Similarities))<br>- Trend in species richness and diversity: EBF <non-EBF<Non-BF<br>Relative abundance:<br>- Bacterial composition: EBF >non- EBF: Actinobacteria (phylum), *Bifidobacterium* (genus)<br>- non- EBF >EBF : Bacteroidetes (phylum), Clostridiales (order), Lachnospiraceae (family),<br>*Blautia* and *Faecalibacterium* (genus)	Assembled 16S reads used for otu-picking (.fna file), mapping file.<br>- Open OTU picking with UCLUST with 97% similarity using the Greengenes database (version 13.8)	The effect of infant age on breastfeeding status and gut microbiome was not accounted (neither in the design nor in analysis).

Studies with three breastfeeding categories (exclusive breastfeeding (EBF), non-exclusive breastfeeding (non-EBF), non-breastfeeding (non-BF)) used for trend tests across three categories.

$ Studies with available birth mode information used for meta-analysis stratified by birth mode.

€ Studies with available infant sex information used for the analyses adjusting for infant age and sex.

* This study contains data from 6 months to 2 years of age, which was used for the analysis from 6 months to 2 years of age. Data from this study was downloaded from the authors' website:

https://gordonlab.wustl.edu/Subramanian_6_14/Nature_2014_Processed_16S_rRNA_datasets.html. Data from six other studies were obtained directly from the investigators.

Supplementary Figures

Supplementary Figure 1. Meta-analysis of included studies without data from either North Carolina, Haiti or VDAART trial study for microbial alpha diversity difference between non-exclusively breastfed vs. exclusively breastfed infants (sensitivity analysis).

a

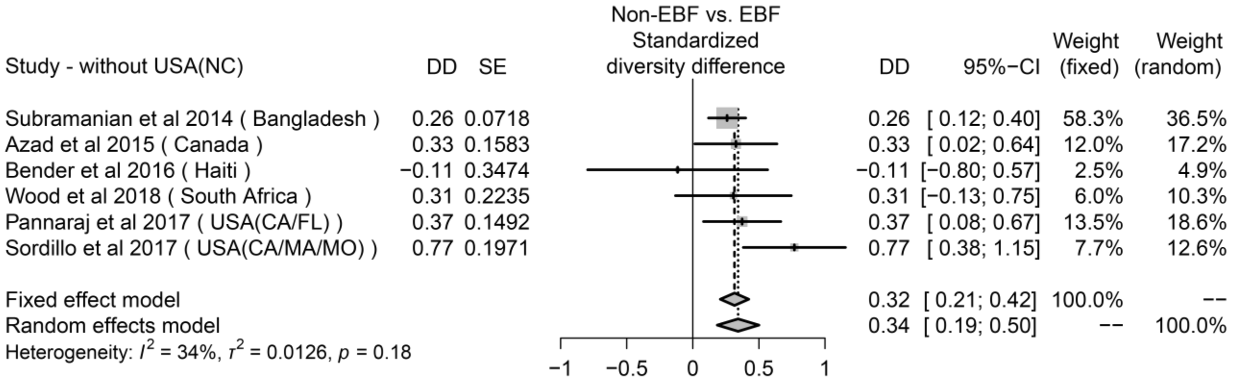

b

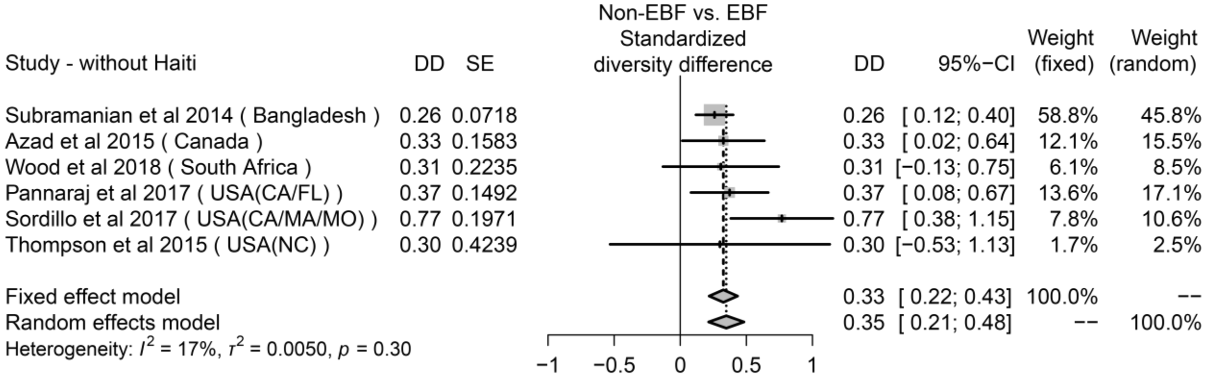

c

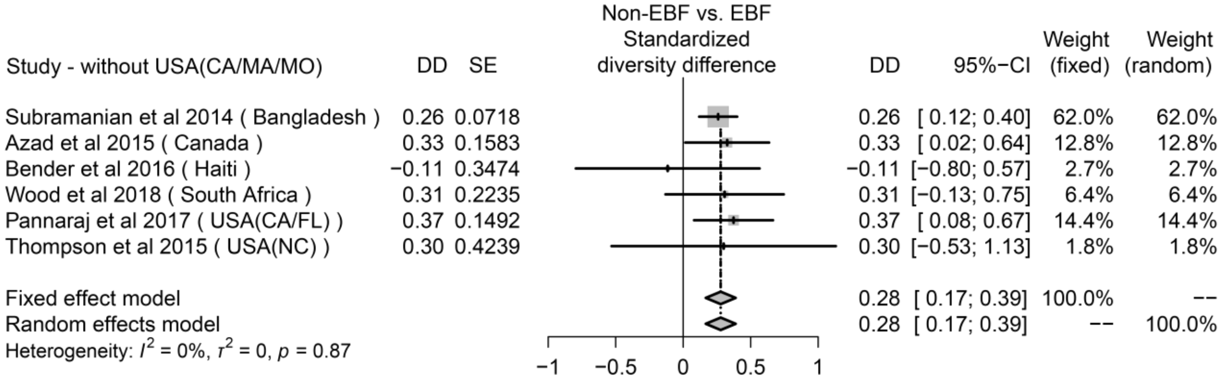

a: Meta-analysis without estimates from the USA (North Carolina) study.

b: Meta-analysis without estimates from the Haiti study.

c: Meta-analysis without estimates from the VDAART trial (USA[California-Massachusetts-Missouri]) study.

The figures show the difference in gut microbial alpha diversity (standardized Shannon index) between non-exclusively breastfed (non-EBF) vs. EBF infants ≤ 6 months of age from each study and the pooled effect across studies with 95% confidence intervals. Estimates for diversity difference and corresponding standard errors from each study were from linear mixed effect models (for longitudinal data) or linear models (for non-longitudinal data) and were adjusted for age of infants at sample collection.

EBF: exclusive breastfeeding; non-EBF: non-exclusive breastfeeding; USA: United States of America; CA: California; FL: Florida; MA: Massachusetts; MO: Missouri; NC: North Carolina; DD: Diversity difference; SE: Standard error; VDAART: Vitamin D Antenatal Asthma Reduction Trial.

Supplementary Figure 2. Meta-analysis stratified by mode of delivery for differences in microbial alpha diversity (Shannon index) between non-exclusively breastfed vs. exclusively breastfed infants ≤6 months of age.

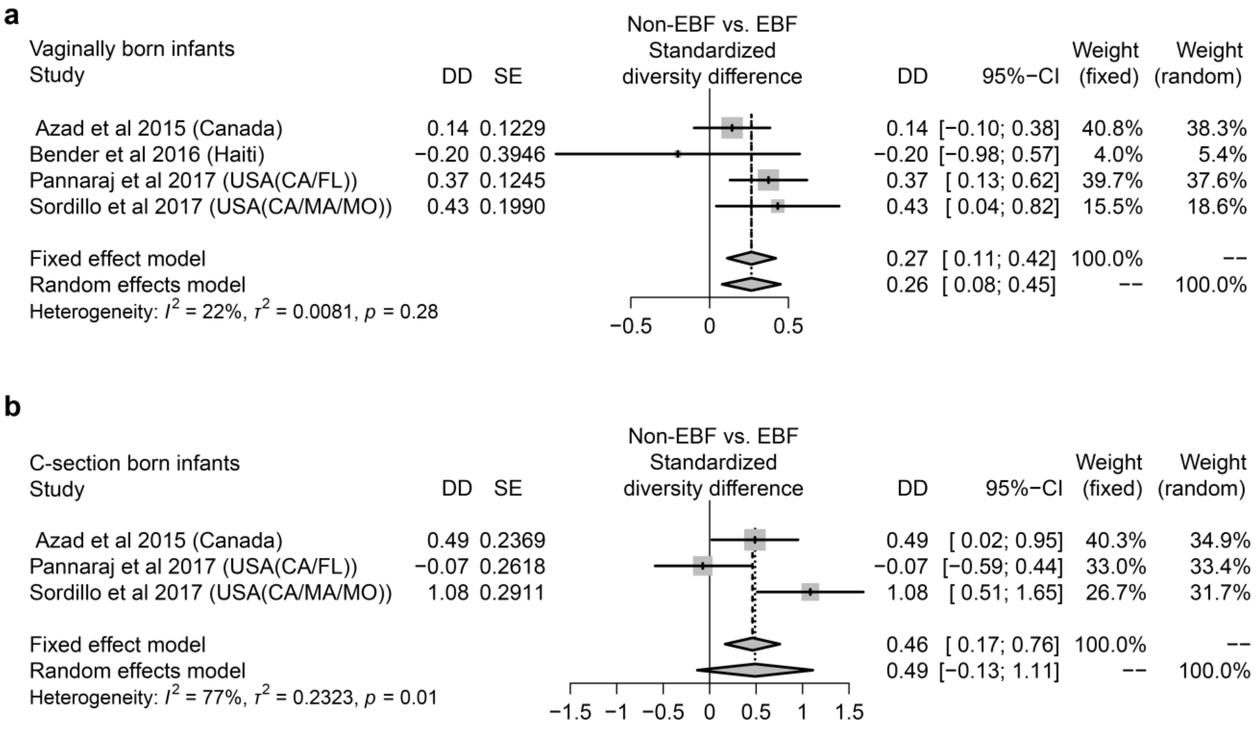

a. Meta-analysis of vaginally delivered infants.

b. Meta-analysis of cesarean delivered infants.

The figures show the difference in gut alpha diversity (standardized Shannon index) between non-exclusively breastfed (non-EBF) vs. EBF infants ≤ 6 months of age from each study and the pooled effect across studies with 95% confidence intervals. Estimates for diversity difference and corresponding standard errors from each study were from linear mixed effect models (for longitudinal data) or linear models (for non-longitudinal data) and were adjusted for age of infants at sample collection.

EBF: exclusive breastfeeding; non-EBF: non-exclusive breastfeeding; USA: United States of America; CA: California; FL: Florida; MA: Massachusetts; MO: Missouri; DD: Diversity difference; SE: Standard error.

Supplementary Figure 3. Analysis adjusting for infant age vs. analysis adjusting for both infant age and sex for differences in microbial alpha diversity indexes between non-exclusively breastfed vs. exclusively breastfed infants ≤6 months of age.

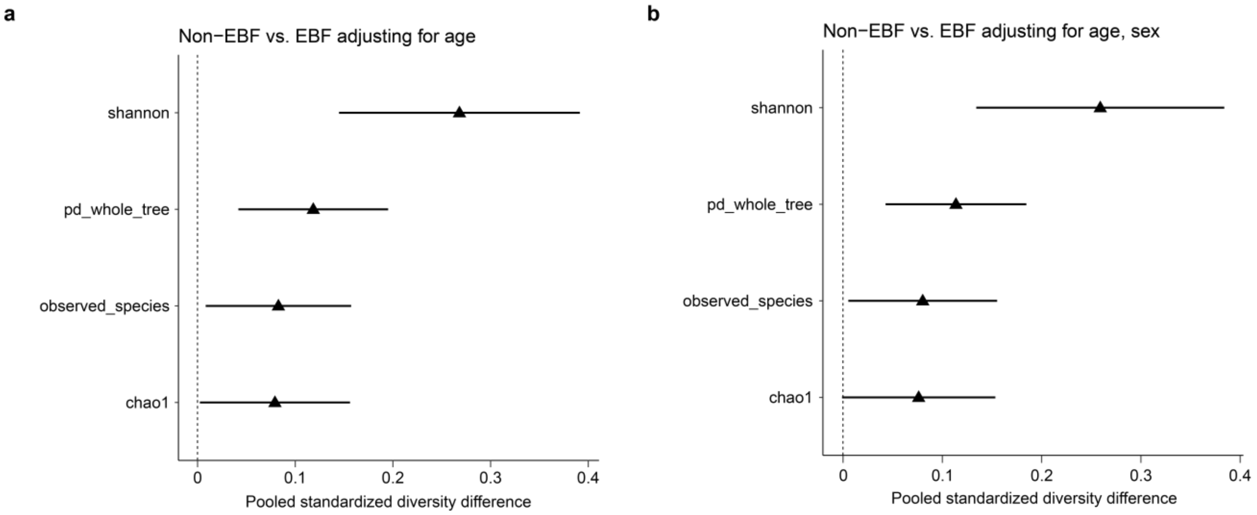

a. Non-exclusive breastfeeding (non-EBF) vs. EBF adjusting for infant age.

b. Non-EBF vs. EBF adjusting for infant age and sex.

The analysis was done in a subset of four studies with available data on infant sex (Bangladesh, Haiti, USA [California-Florida], USA [North Carolina]).

Estimates for diversity difference and corresponding standard errors from each study were from linear mixed effect models (for longitudinal data) or linear models (for non-longitudinal data) and were adjusted for infant age at sample collection (a) or adjusted for infant age at sample collection and infant sex (b). Pooled estimates of standardized diversity difference and their 95% confidence intervals were from random effect meta-analysis models based on the adjusted estimates and corresponding standard errors of all included studies.

Supplementary Figure 4. Performance of Random Forest model in prediction of gut microbiota age on the training and test set of Bangladesh data.

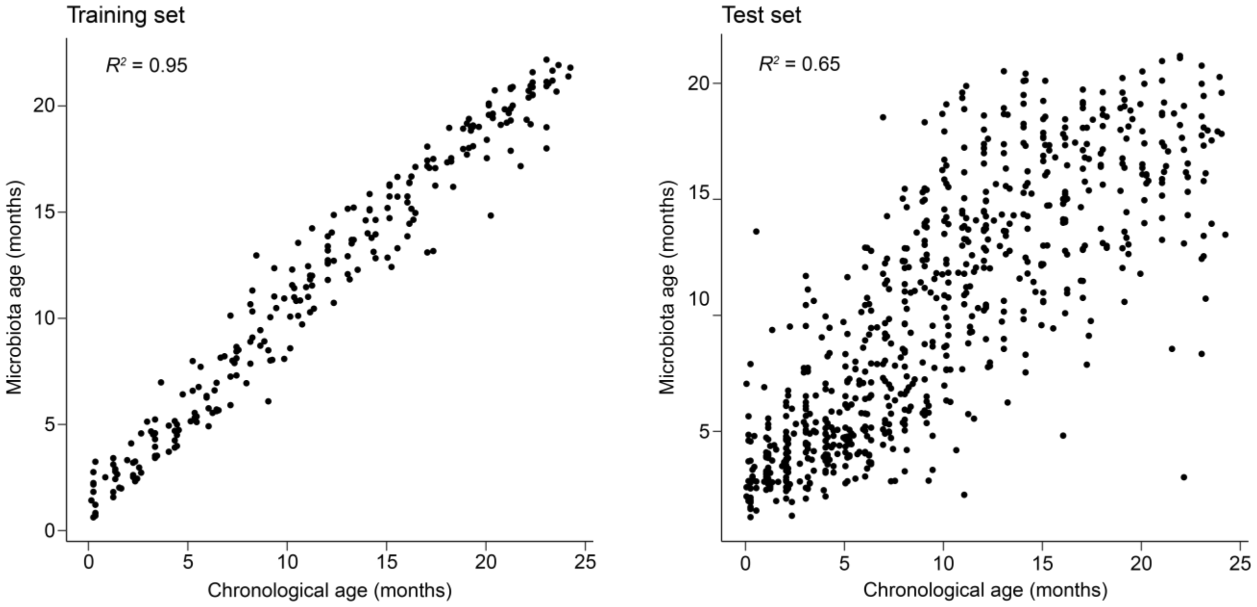

Supplementary Figure 5. Meta-analysis of included studies without data from either North Carolina, Haiti or VDAART trial study for microbiota age difference between non-exclusively breastfed vs. exclusively breastfed infants (sensitivity analysis).

a

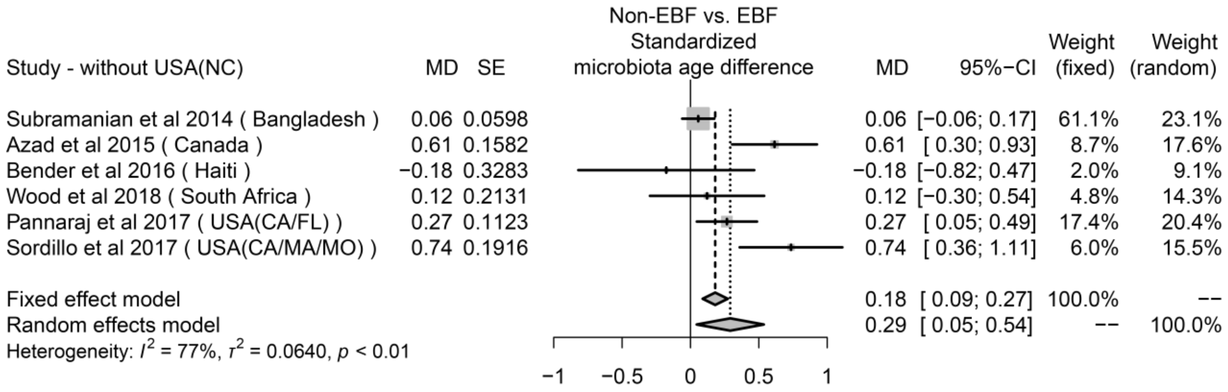

b

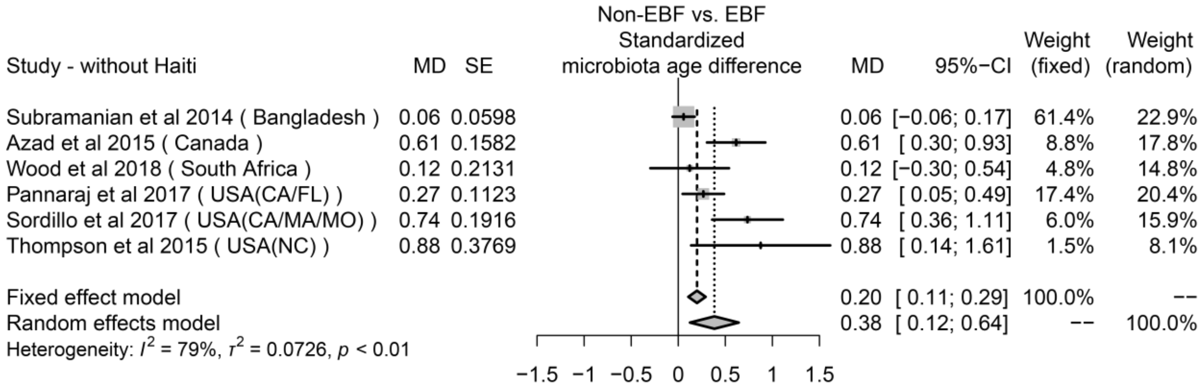

c

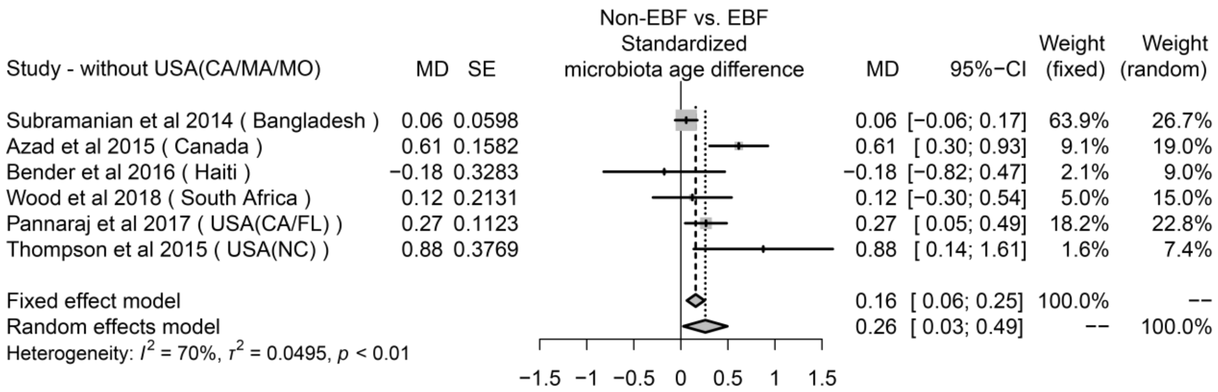

a: Meta-analysis without estimates from North Carolina study.

b: Meta-analysis without estimates from Haiti study.

c: Meta-analysis without estimates from VDAART trial (USA[California-Massachusetts-Missouri]) study.

The figures show the difference in gut (standardized) microbiota age between non-exclusively breastfed (non-EBF) vs. EBF infants ≤ 6 months of age from each study and the pooled effect across studies with 95% confidence intervals. Estimates for microbiota age difference and corresponding standard error from each study were from linear mixed effect models (for longitudinal data) or linear models (for non-longitudinal data) and were adjusted for age of infants at sample collection.

EBF: exclusive breastfeeding; non-EBF: non-exclusive breastfeeding; USA: United States of America; CA: California; FL: Florida; MA: Massachusetts; MO: Missouri; NC: North Carolina; MD: Microbiota age difference; SE: Standard error; VDAART: Vitamin D Antenatal Asthma Reduction Trial.

Supplementary Figure 6. Meta-analysis stratified by mode of delivery for differences in microbiota age between non-exclusively breastfed vs. exclusively breastfed infants ≤6 months of age.

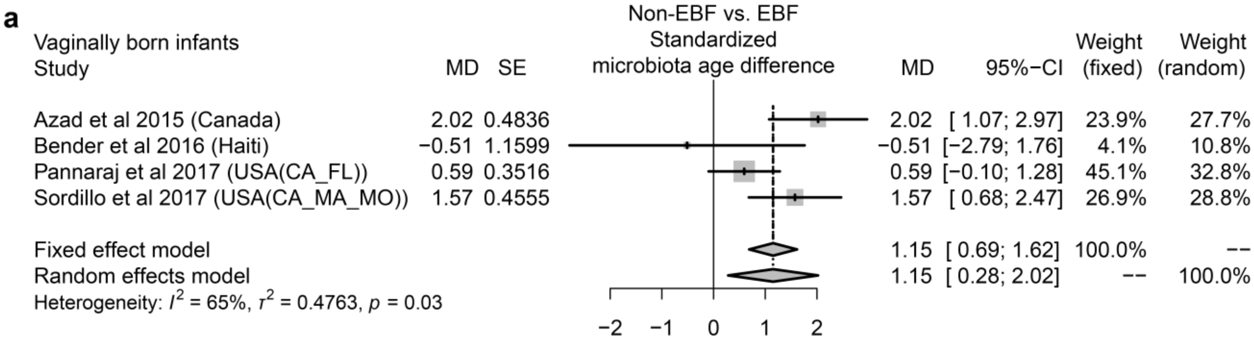

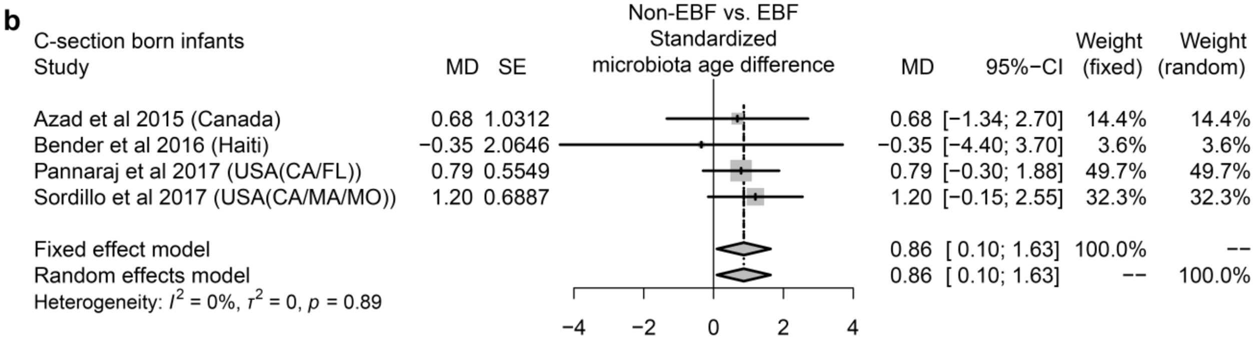

a. Meta-analysis of vaginally born infants only.

b. Meta-analysis of C-section born infants only.

The figures show the difference in gut microbiota age between non-exclusively breastfed (non-EBF) vs. EBF infants ≤ 6 months of age from each study and the pooled effect (meta-analysis) with 95% confidence intervals across four studies with available mode of delivery information. Estimates for microbiota age difference and corresponding standard error from each study were from linear mixed effect models (for longitudinal data) or linear models (for non-longitudinal data) and were adjusted for age of infants at sample collection.

EBF: exclusive breastfeeding; non-EBF: non-exclusive breastfeeding; USA: United States of America; CA: California; FL: Florida; MA: Massachusetts; MO: Missouri; MD: Microbiota age difference; SE: Standard error.

Supplementary Figure 7. Analysis adjusting for infant age vs. analysis adjusting for both infant age and sex for differences in microbiota age between non-exclusively breastfed vs. exclusively breastfed infants ≤6 months of age.

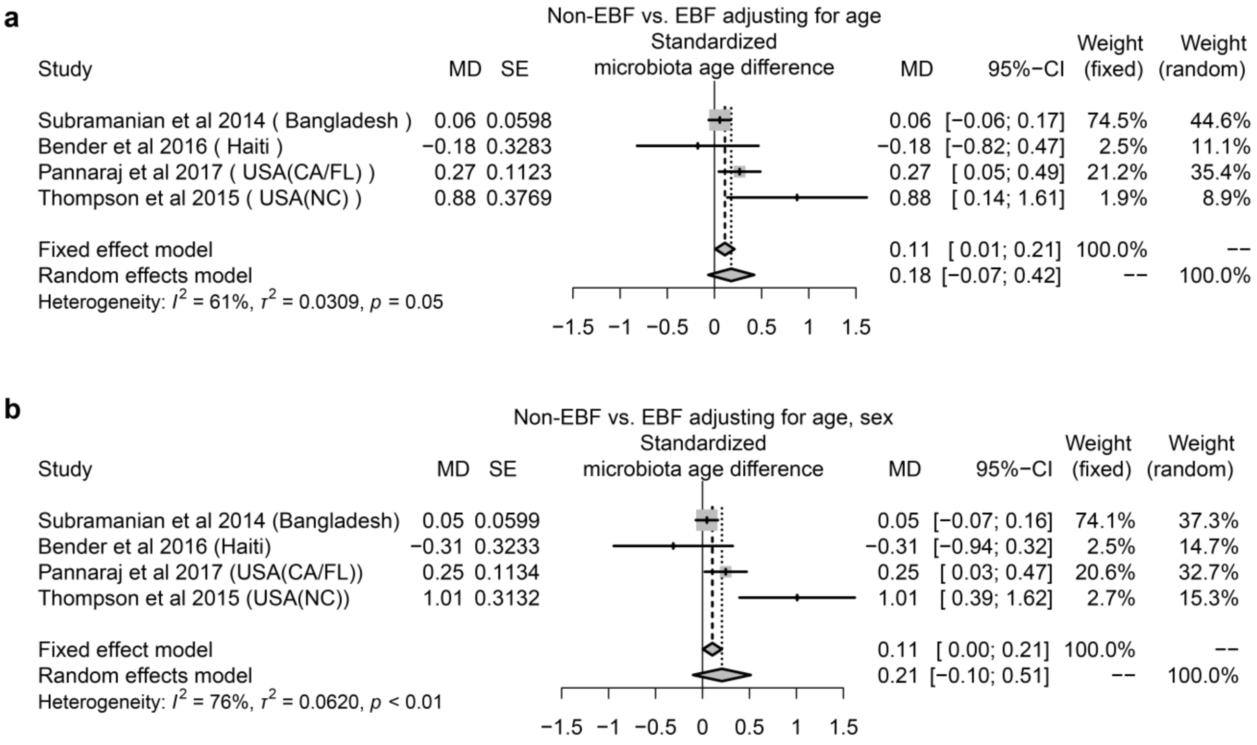

a. Non-exclusive breastfeeding (non-EBF) vs. EBF adjusting for infant age.

b. Non-EBF vs. EBF adjusting for infant age and sex.

The figures show the difference in gut microbiota age between non-exclusively breastfed (non-EBF) vs. EBF infants ≤ 6 months of age from each study and the pooled effect (meta-analysis) with 95% confidence intervals across four studies with available infant sex information. Estimates for microbiota age difference and corresponding standard error from each study were from linear mixed effect models (for longitudinal data) or linear models (for non-longitudinal data) and were adjusted for infant age at sample collection (a) or adjusted for both infant age at sample collection and infant sex (b).

EBF: exclusive breastfeeding; non-EBF: non-exclusive breastfeeding; USA: United States of America; CA: California; FL: Florida; NC: North Carolina; MD: Microbiota age difference; SE: Standard error.

Supplementary Figure 8. Meta-analysis of seven included studies for the effects of non-exclusive vs. exclusive breastfeeding on relative abundance of gut bacterial taxa at order level in infants ≤ 6 months.

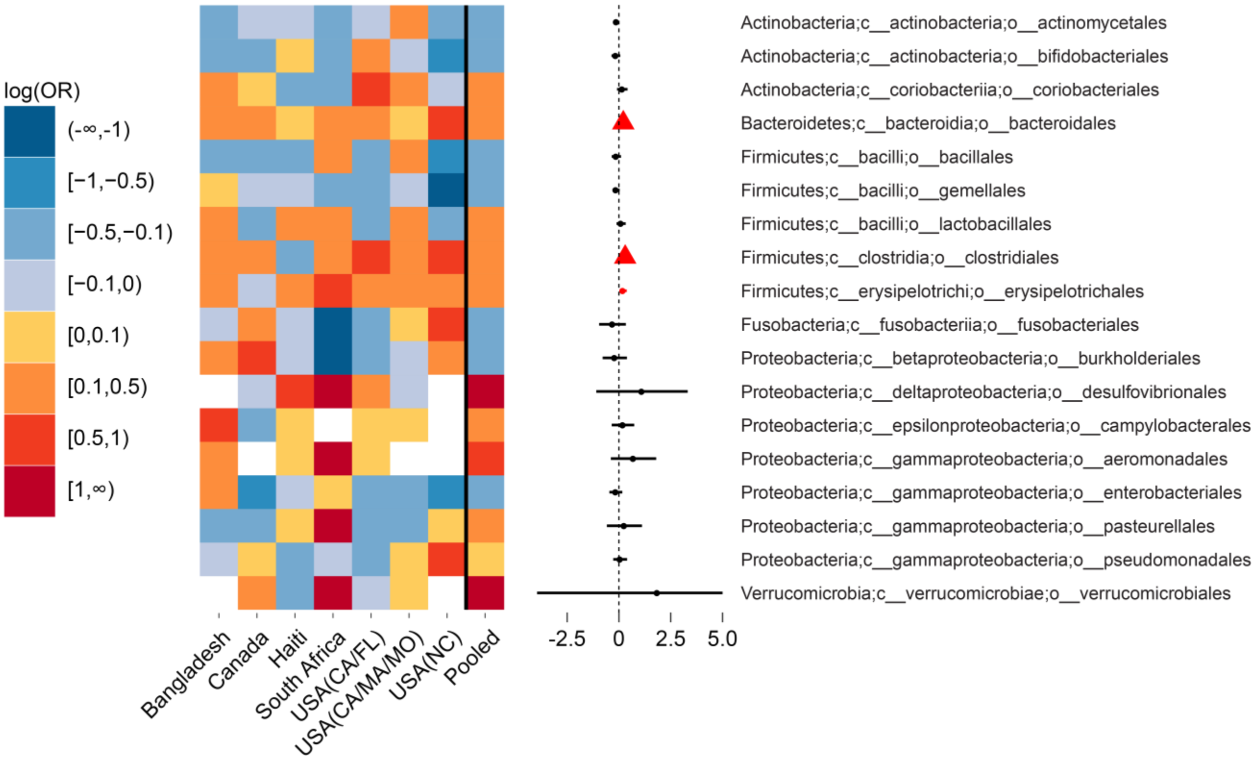

Heatmap of log(odds ratio) (log(OR)) of relative abundance of all gut bacterial taxa at order level between non-exclusively breastfed (non-EBF) vs. exclusively breastfed (EBF) infants for each study and forest plot of pooled estimates of all studies with 95% confidence intervals (95% CI). All log(OR) estimates of each order from each study were from Generalized Additive Models for Location Scale and Shape (GAMLSS) with beta zero inflated family (BEZI) and were adjusted for age of infants at sample collection. Pooled log(OR) estimates and 95% CI (forest plot) were from random effect meta-analysis models based on the adjusted log(OR) estimates and corresponding standard errors of all included studies.

Pooled log(OR) estimates with pooled p-values<0.05 are in red and those with false discovery rate (FDR) adjusted pooled p-values <0.1 are shown as triangles.

Missing (unavailable) values are in white.

USA: United States of America; CA: California; FL: Florida; MA: Massachusetts; MO: Missouri; NC: North Carolina.

Supplementary Figure 9. Meta-analysis of seven included studies for the effects of non-exclusive vs. exclusive breastfeeding on relative abundance of gut bacterial taxa at genus level in infants ≤ 6 months.

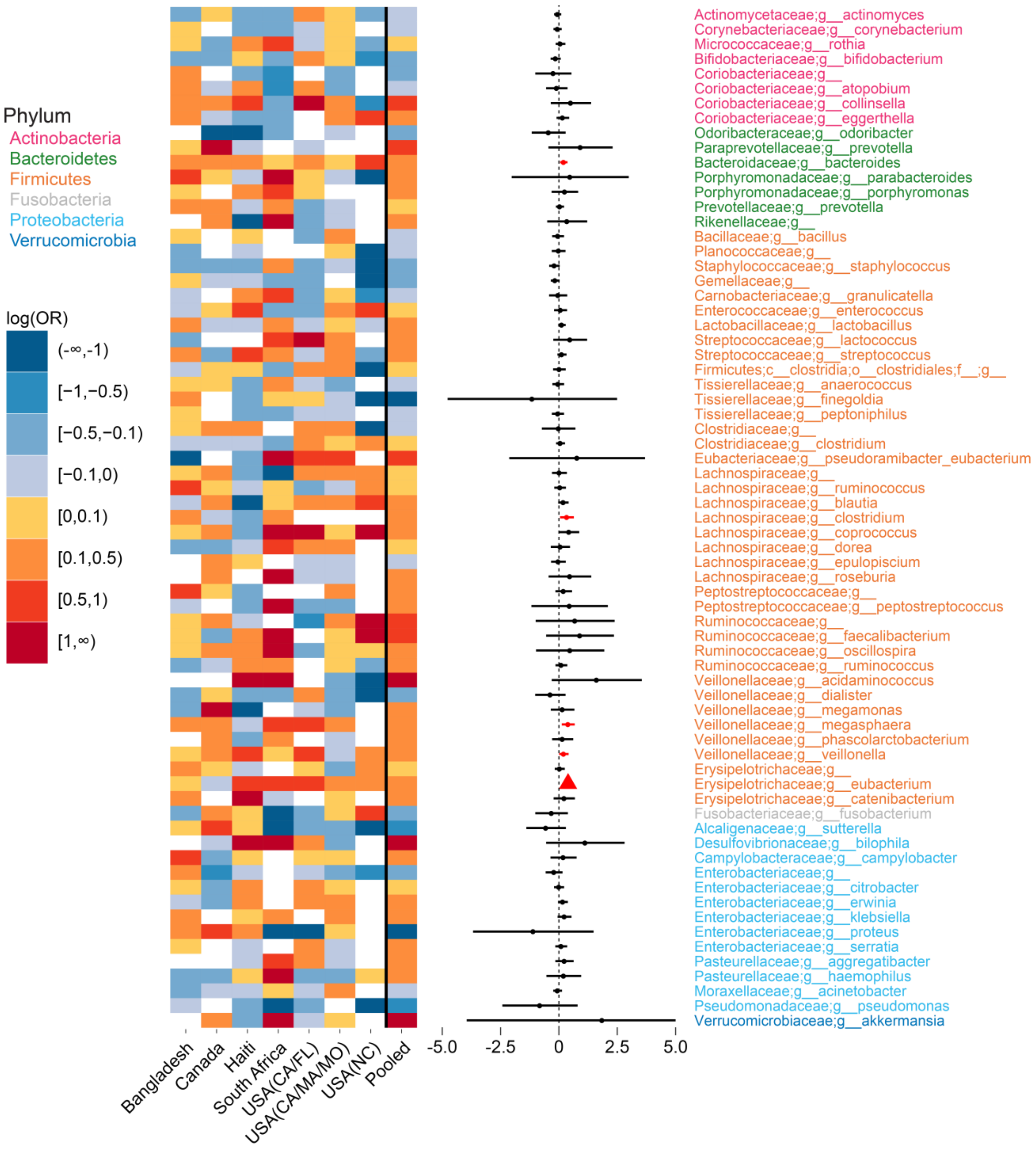

Heatmap of log(odds ratio) (log(OR)) of relative abundance of all gut bacterial taxa at genus level between non-exclusively breastfed (non-EBF) vs. exclusively breastfed (EBF) infants for each study and forest plot of pooled estimates of all studies with 95% confidence intervals (95% CI). All log(OR) estimates of each genus from each study were from Generalized Additive Models for Location Scale and Shape (GAMLSS) with beta zero inflated family (BEZI) and were adjusted for age of infants at sample collection. Pooled log(OR) estimates and 95% CI (forest plot) were from random effect meta-analysis models based on the adjusted log(OR) estimates and corresponding standard errors of all included studies.

Pooled log(OR) estimates with pooled p-values<0.05 are in red and those with false discovery rate (FDR) adjusted pooled p-values <0.1 are shown as triangles.

Missing (unavailable) values are in white.

USA: United States of America; CA: California; FL: Florida; MA: Massachusetts; MO: Missouri; NC: North Carolina.

Supplementary Figure 10. Meta-analysis stratified by mode of delivery for the effects of non-exclusive vs. exclusive breastfeeding on relative abundance of gut bacterial taxa at phylum level in infants ≤ 6 months.

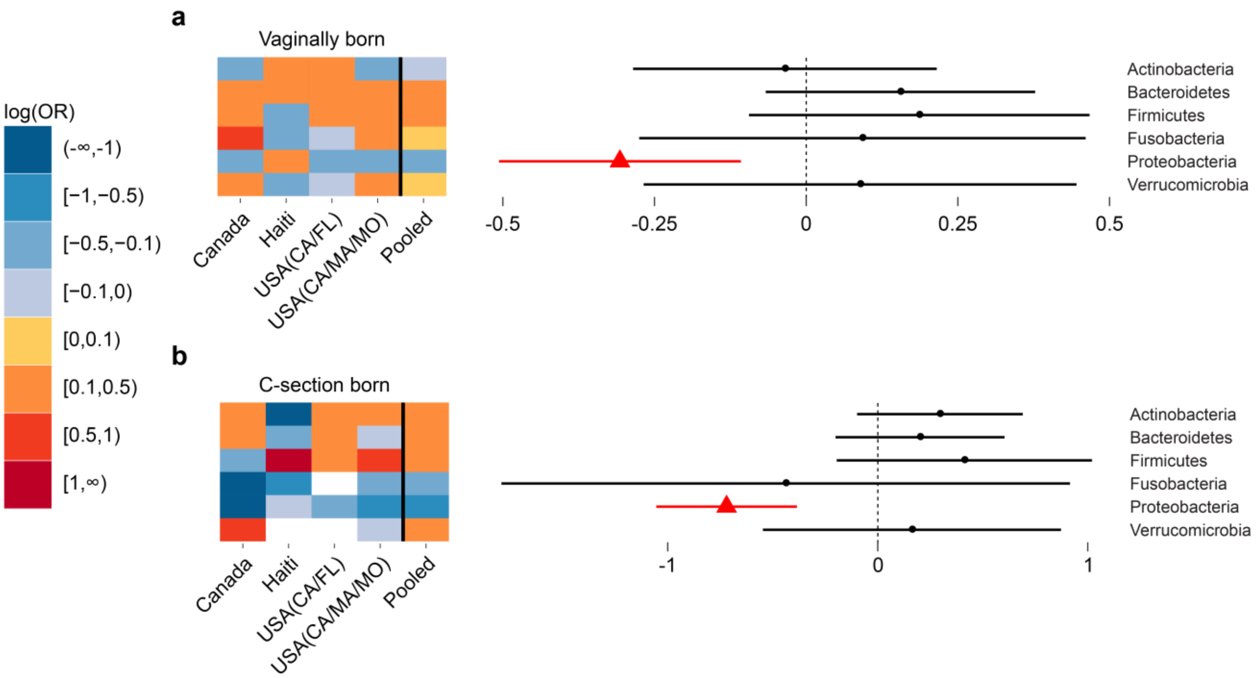

a. Meta-analysis of vaginally born infants only.

b. Meta-analysis of C-section born infants only.

The figures show heatmap of log(odds ratio) (log(OR)) of relative abundances of all gut bacterial phyla between non-exclusively breastfed (non-EBF) vs. exclusively breastfed (EBF) infants for each study and forest plot of pooled estimates across four studies (with available mode of delivery information) and 95% confidence intervals (95% CI).

All log(OR) estimates of each bacterial phylum from each study were from Generalized Additive Models for Location Scale and Shape (GAMLSS) with beta zero inflated family (BEZI) and were adjusted for age of infants at sample collection. Pooled log(OR) estimates and 95% CI (forest plot) were from random effect meta-analysis models based on the adjusted log(OR) estimates and corresponding standard errors of all included studies.

Pooled log(OR) estimates with pooled p-values<0.05 are in red and those with false discovery rate (FDR) adjusted pooled p-values <0.1 are shown as triangles.

Missing (unavailable) values are in white.

USA: United States of America; CA: California; FL: Florida; MA: Massachusetts; MO: Missouri.

Supplementary Figure 11. Meta-analysis stratified on vaginally delivered infants for the effects of non-exclusive vs. exclusive breastfeeding on relative abundance of gut bacterial taxa at family level in infants ≤ 6 months.

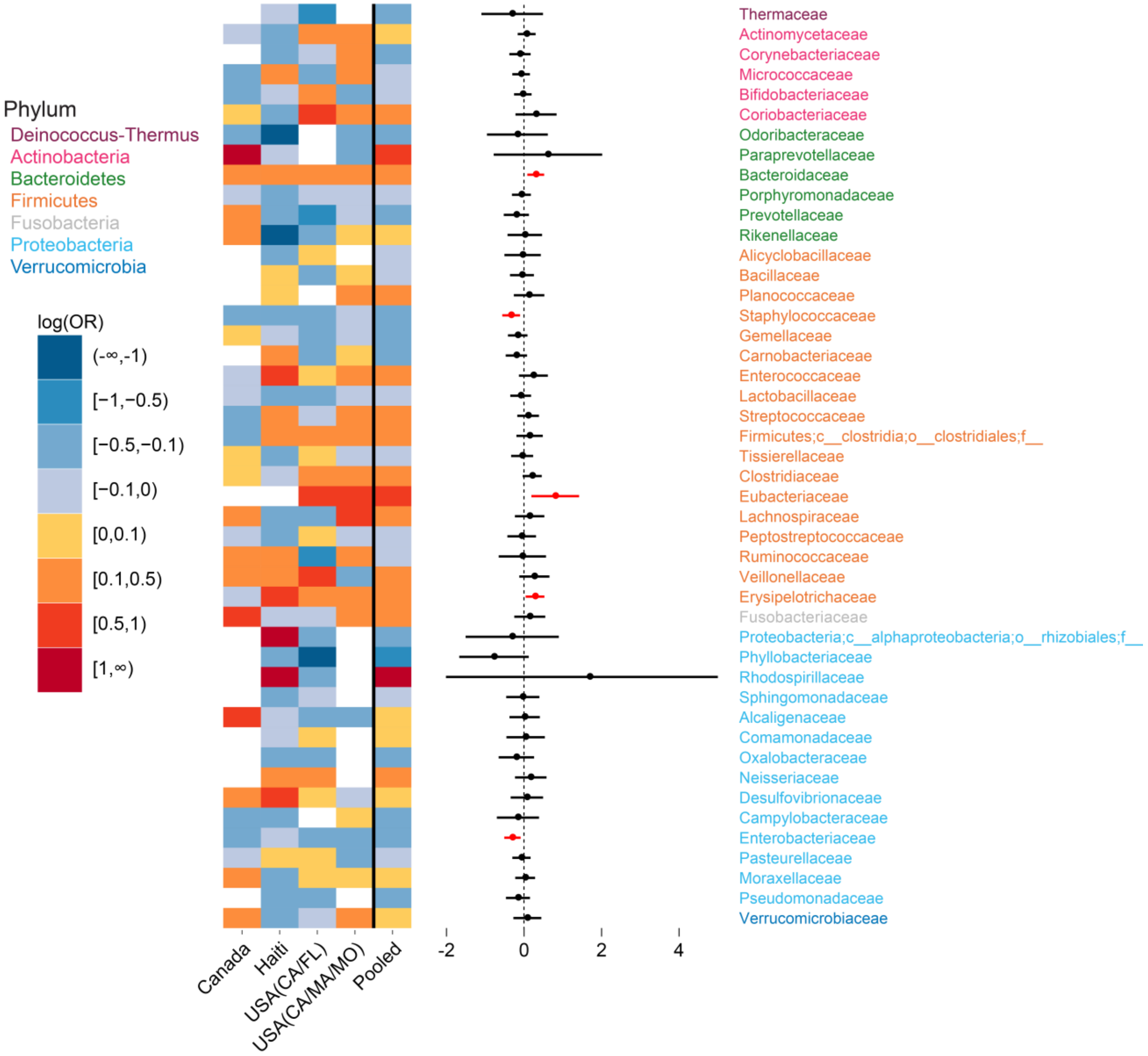

Heatmap of log(odds ratio) (log(OR)) of relative abundances of all gut bacterial families between non-exclusively breastfed (non-EBF) vs. exclusively breastfed (EBF) infants for each study and forest plot of pooled estimates across four studies (with available mode of delivery information) and 95% confidence intervals (95% CI).

All log(OR) estimates of each bacterial taxa from each study were from Generalized Additive Models for Location Scale and Shape (GAMLSS) with beta zero inflated family (BEZI) and were adjusted for age of infants at sample collection. Pooled log(OR) estimates and 95% CI (forest plot) were from random effect meta-analysis models based on the adjusted log(OR) estimates and corresponding standard errors of all included studies.

Pooled log(OR) estimates with pooled p-values<0.05 are in red and those with false discovery rate (FDR) adjusted pooled p-values <0.1 are shown as triangles.

Missing (unavailable) values are in white.

USA: United States of America; CA: California; FL: Florida; MA: Massachusetts; MO: Missouri.

Supplementary Figure 12. Meta-analysis stratified on C-section delivered infants for the effects of non-exclusive vs. exclusive breastfeeding on relative abundance of gut bacterial taxa at family level in infants ≤ 6 months.

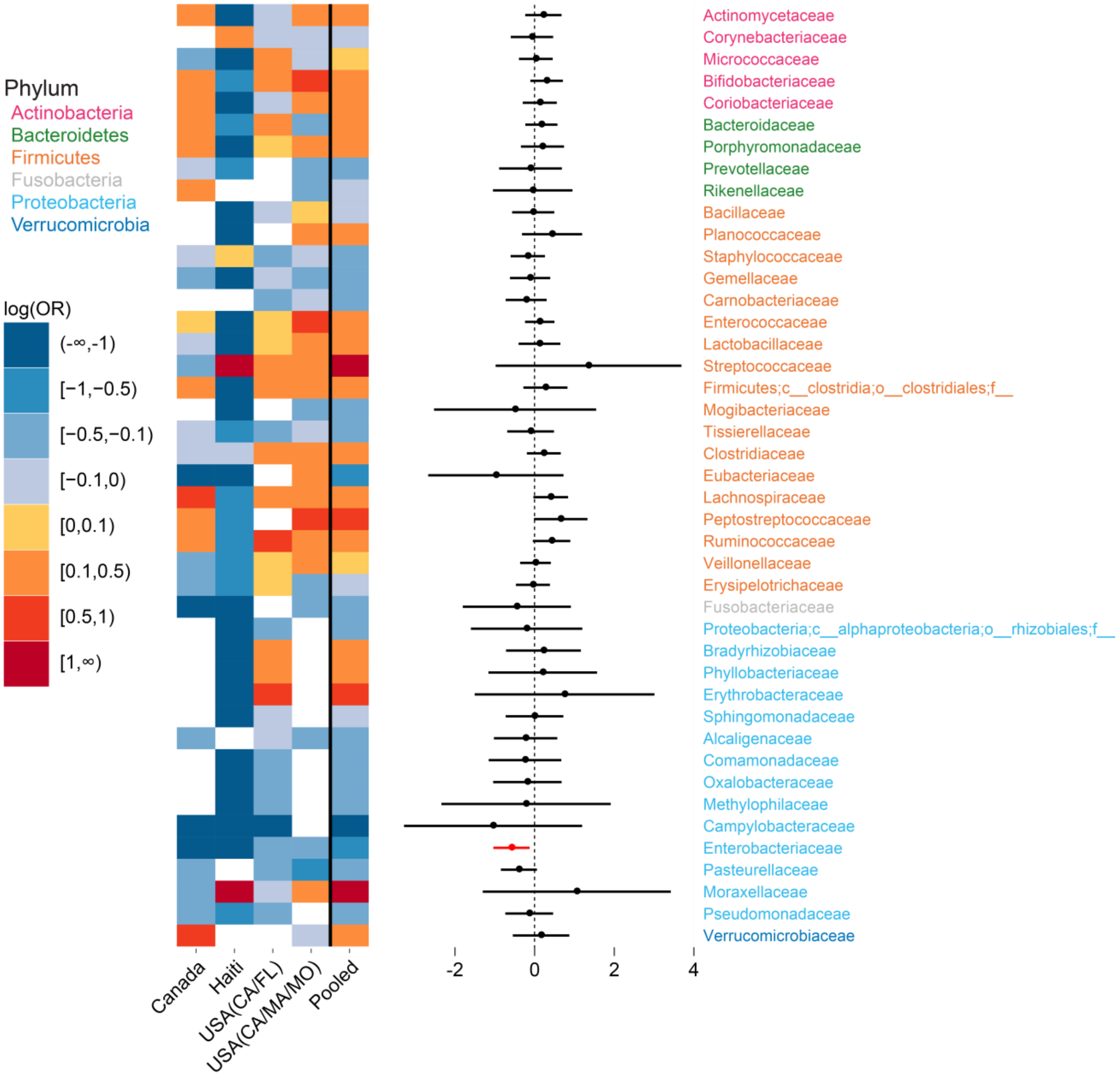

Heatmap of log(odds ratio) (log(OR)) of relative abundances of all gut bacterial families between non-exclusively breastfed (non-EBF) vs. exclusively breastfed (EBF) infants for each study and forest plot of pooled estimates across four studies (with available mode of delivery information) and 95% confidence intervals (95% CI).

All log(OR) estimates of each bacterial taxa from each study were from Generalized Additive Models for Location Scale and Shape (GAMLSS) with beta zero inflated family (BEZI) and were adjusted for age of infants at sample collection. Pooled log(OR) estimates and 95% CI (forest plot) were from random effect meta-analysis models based on the adjusted log(OR) estimates and corresponding standard errors of all included studies.

Pooled log(OR) estimates with pooled p-values<0.05 are in red and those with false discovery rate (FDR) adjusted pooled p-values <0.1 are shown as triangles.

Missing (unavailable) values are in white.

USA: United States of America; CA: California; FL: Florida; MA: Massachusetts; MO: Missouri.

Supplementary Figure 13. Meta-analysis stratified on vaginally delivered infants for the effects of non-exclusive vs. exclusive breastfeeding on relative abundance of gut bacterial taxa at genus level in infants ≤ 6 months.

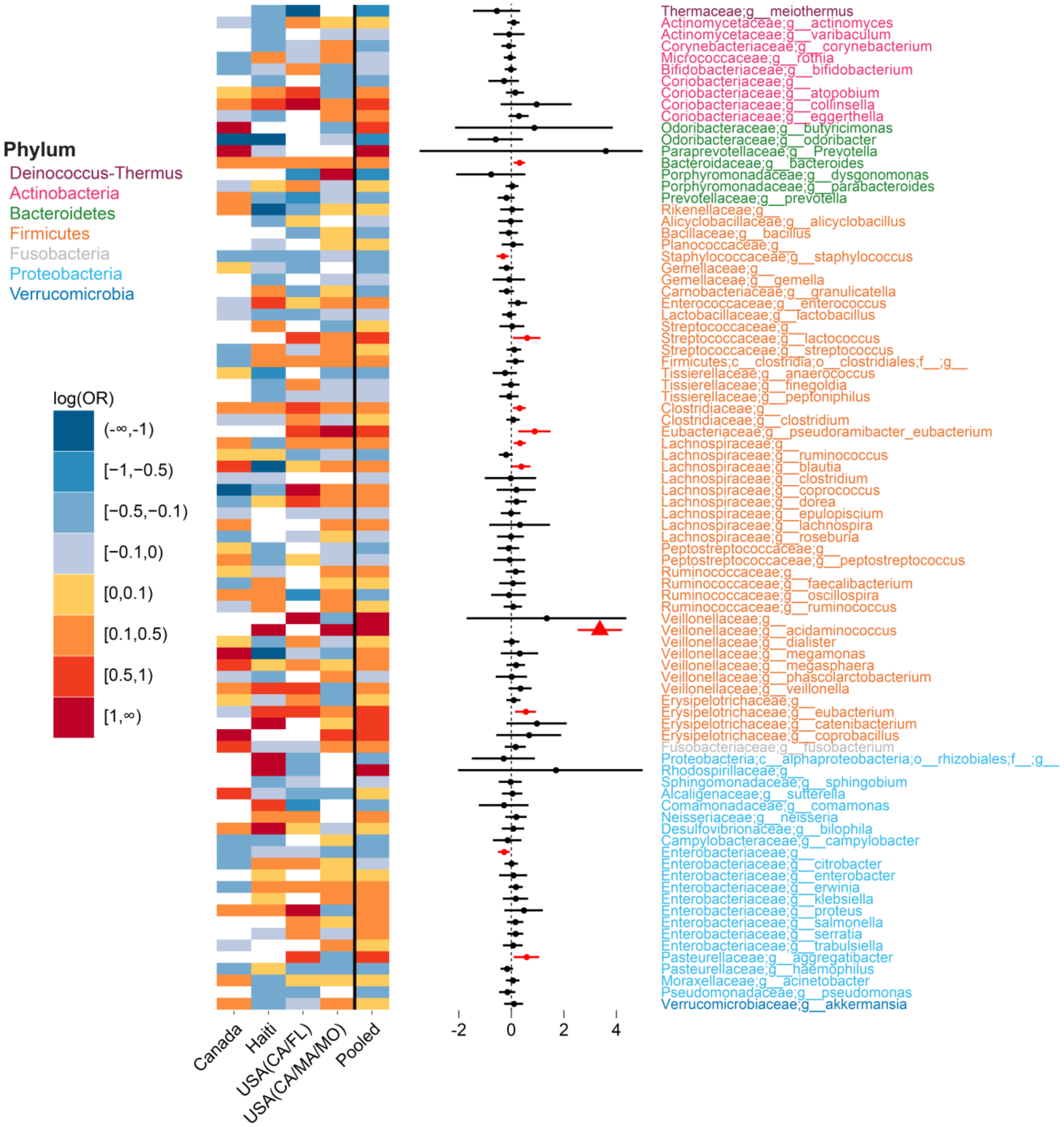

Heatmap of log(odds ratio) (log(OR)) of relative abundances of all gut bacterial genera between non-exclusively breastfed (non-EBF) vs. exclusively breastfed (EBF) infants for each study and

forest plot of pooled estimates across four studies (with available mode of delivery information) and 95% confidence intervals (95% CI).

All log(OR) estimates of each bacterial taxa from each study were from Generalized Additive Models for Location Scale and Shape (GAMLSS) with beta zero inflated family (BEZI) and were adjusted for age of infants at sample collection. Pooled log(OR) estimates and 95% CI (forest plot) were from random effect meta-analysis models based on the adjusted log(OR) estimates and corresponding standard errors of all included studies.

Pooled log(OR) estimates with pooled p-values<0.05 are in red and those with false discovery rate (FDR) adjusted pooled p-values <0.1 are shown as triangles.

Missing (unavailable) values are in white.

USA: United States of America; CA: California; FL: Florida; MA: Massachusetts; MO: Missouri.

Supplementary Figure 14. Meta-analysis stratified on C-section delivered infants for the effects of non-exclusive vs. exclusive breastfeeding on relative abundance of gut bacterial taxa at genus level in infants ≤ 6 months.

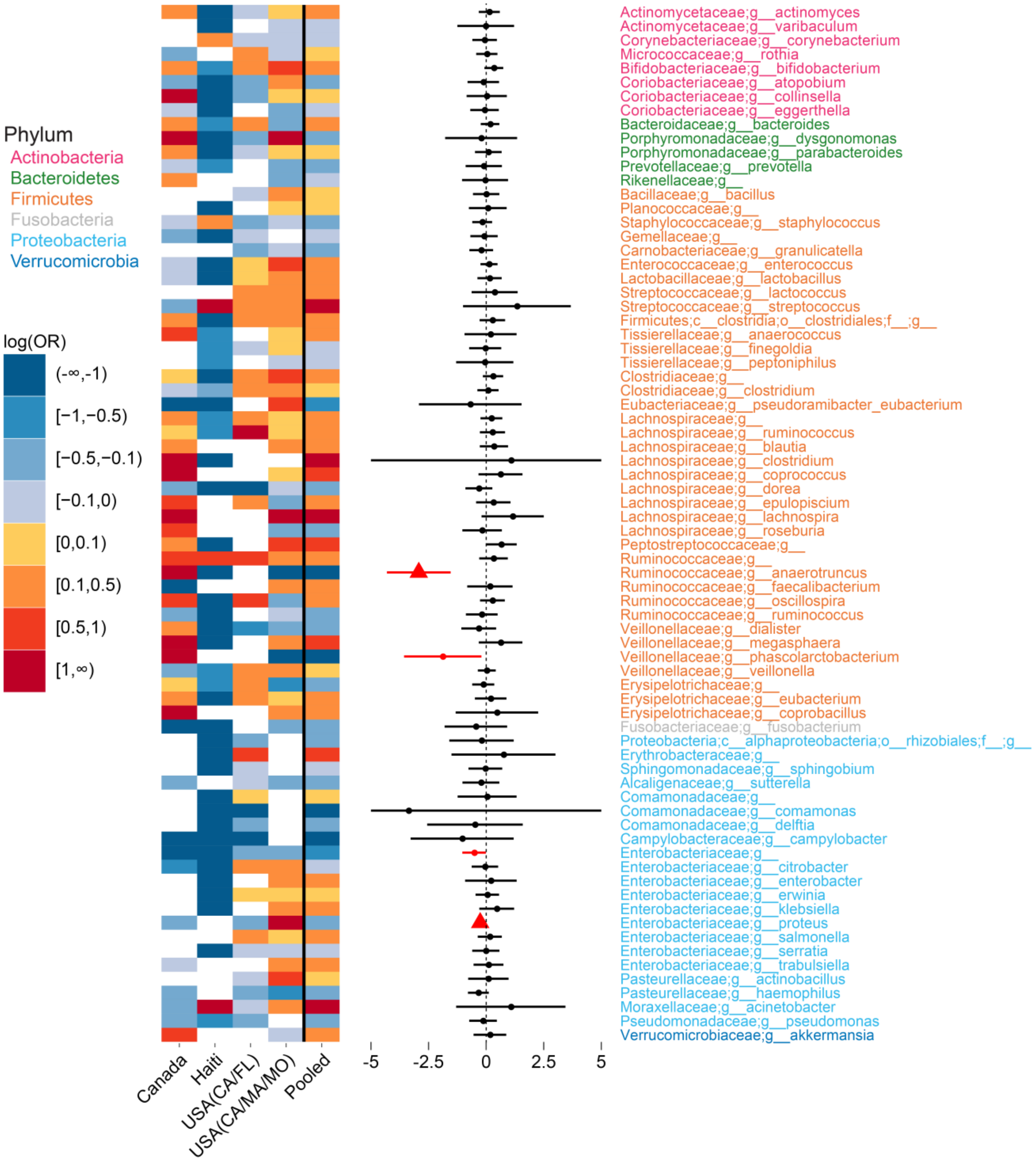

Heatmap of log(odds ratio) (log(OR)) of relative abundances of all gut bacterial genera between non-exclusively breastfed (non-EBF) vs. exclusively breastfed (EBF) infants for each study and forest plot of pooled estimates across four studies (with available mode of delivery information) and 95% confidence intervals (95% CI).

All log(OR) estimates of each bacterial taxa from each study were from Generalized Additive Models for Location Scale and Shape (GAMLSS) with beta zero inflated family (BEZI) and were adjusted for age of infants at sample collection. Pooled log(OR) estimates and 95% CI (forest plot) were from random effect meta-analysis models based on the adjusted log(OR) estimates and corresponding standard errors of all included studies.

Pooled log(OR) estimates with pooled p-values<0.05 are in red and those with false discovery rate (FDR) adjusted pooled p-values <0.1 are shown as triangles.

Missing (unavailable) values are in white.

USA: United States of America; CA: California; FL: Florida; MA: Massachusetts; MO: Missouri.

Supplementary Figure 15. Analysis adjusting for infant age vs. analysis adjusting for both infant age and sex for the effects of non-exclusive vs. exclusive breastfeeding on relative abundance of gut bacterial taxa at phylum level in infants ≤ 6 months.

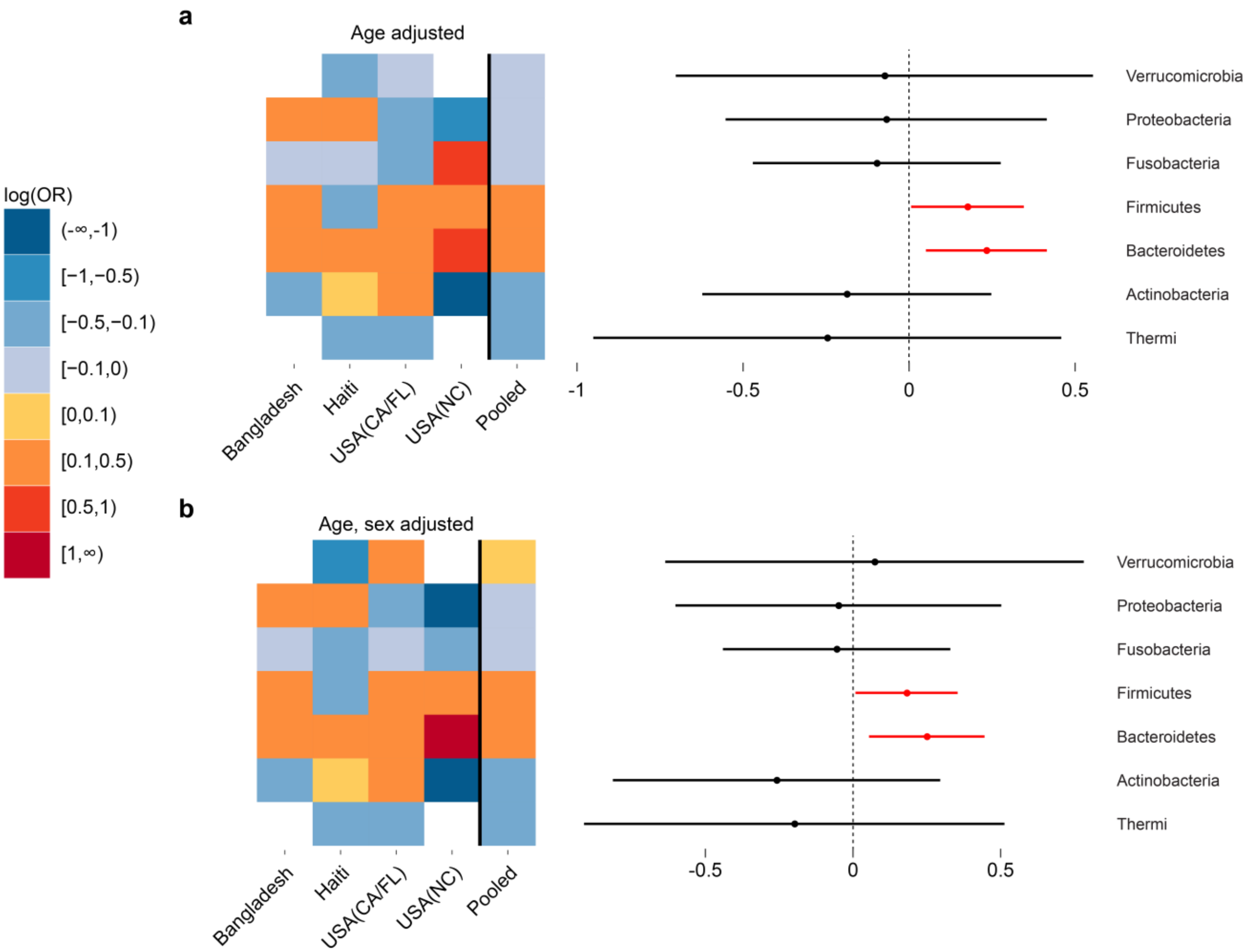

a. Non-exclusive breastfeeding (non-EBF) vs. EBF adjusting for infant age.

b. Non-EBF vs. EBF adjusting for infant age and sex.

The analysis was done in a subset of four studies with available data on infant sex (Bangladesh, Haiti, USA [California-Florida], USA [North Carolina]).

The figures show heatmap of log(odds ratio) (log(OR)) of relative abundances of all gut bacterial phyla between non-exclusively breastfed (non-EBF) vs. exclusively breastfed (EBF) infants for

each study and forest plot of pooled estimates across four studies with 95% confidence intervals (95% CI).

All log(OR) estimates of each bacterial phylum from each study were from Generalized Additive Models for Location Scale and Shape (GAMLSS) with zero inflated beta family (BEZI) and were adjusted for infant age at sample collection (a) or adjusted for both infant age at sample collection and infant sex (b). Pooled log(OR) estimates and 95% CI (forest plot) were from random effect meta-analysis models based on the adjusted log(OR) estimates and corresponding standard errors of all included studies. Pooled log(OR) estimates with pooled p-values<0.05 are in red and those with false discovery rate (FDR) adjusted pooled p-values <0.1 are shown as triangles. Missing (unavailable) values are in white.

USA: United States of America; CA: California; FL: Florida; NC: North Carolina.

Supplementary Figure 16. Meta-analysis of seven included studies for the effects of non-exclusive vs. exclusive breastfeeding on relative abundances of gut microbial KEGG pathways at level two in infants ≤ 6 months.

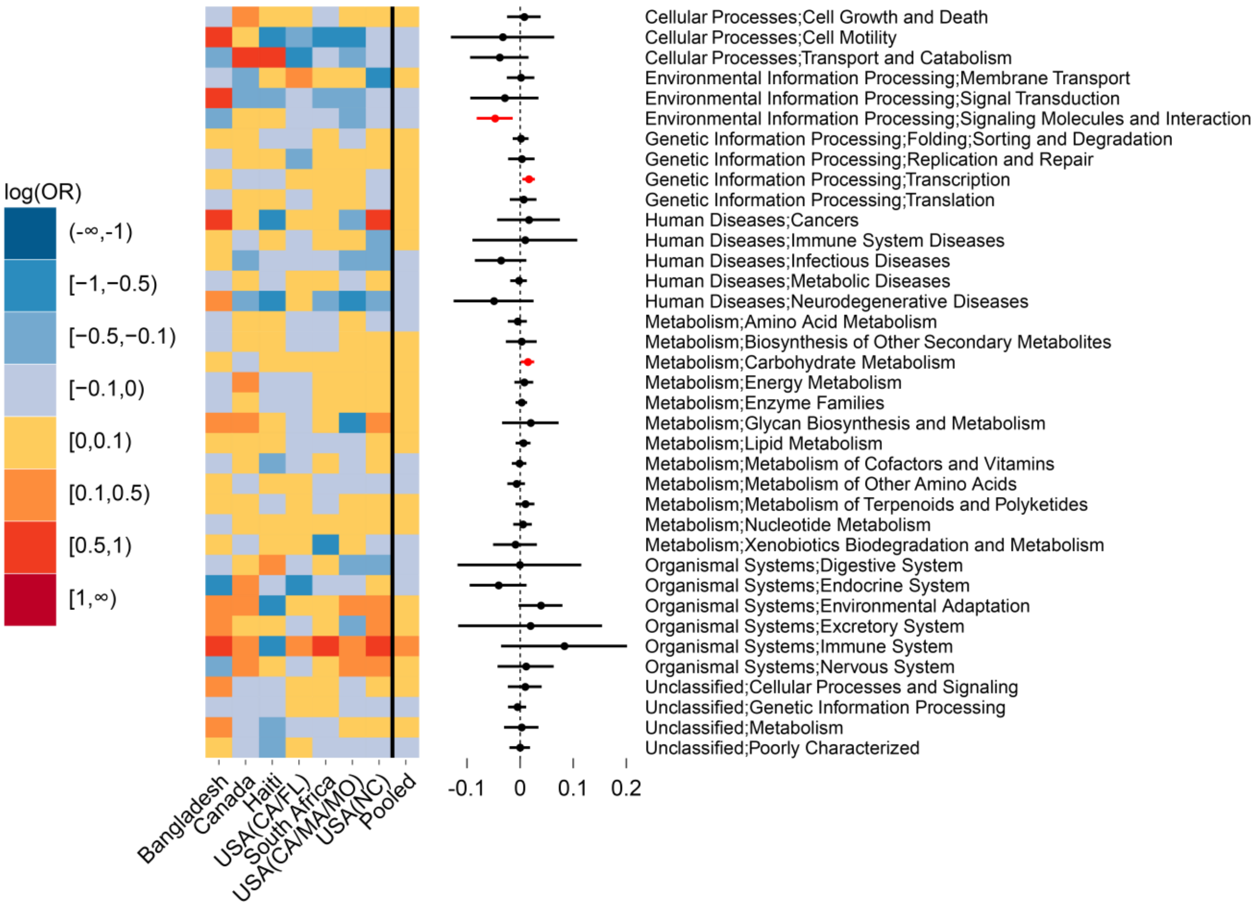

Heatmap of log(odds ratio) (log(OR)) of relative abundances of all gut microbial KEGG pathways at level 2 between non-exclusively breastfed (non-EBF) vs. exclusively breastfed (EBF) infants for each study and forest plot of pooled estimates of all studies with 95% confidence intervals (95% CI). All log(OR) estimates of each pathway from each study were from Generalized Additive Models for Location Scale and Shape (GAMLSS) with beta zero inflated family (BEZI) and were adjusted for age of infants at sample collection. Pooled log(OR) estimates and 95% CI (forest plot) were from random effect meta-analysis models based on the adjusted log(OR) estimates and corresponding standard errors of all included studies.

Pooled log(OR) estimates with pooled p-values<0.05 are in red and those with false discovery rate (FDR) adjusted pooled p-values <0.1 are shown as triangles.

KEGG: Kyoto Encyclopedia of Genes and Genomes; USA: United States of America; CA: California; FL: Florida; MA: Massachusetts; MO: Missouri; NC: North Carolina.

Supplementary Figure 17. Analysis adjusting for infant age vs. analysis adjusting for both infant age and sex for the effects of non-exclusive vs. exclusive breastfeeding on relative abundances of gut microbial KEGG pathways at level three in infants ≤ 6 months.

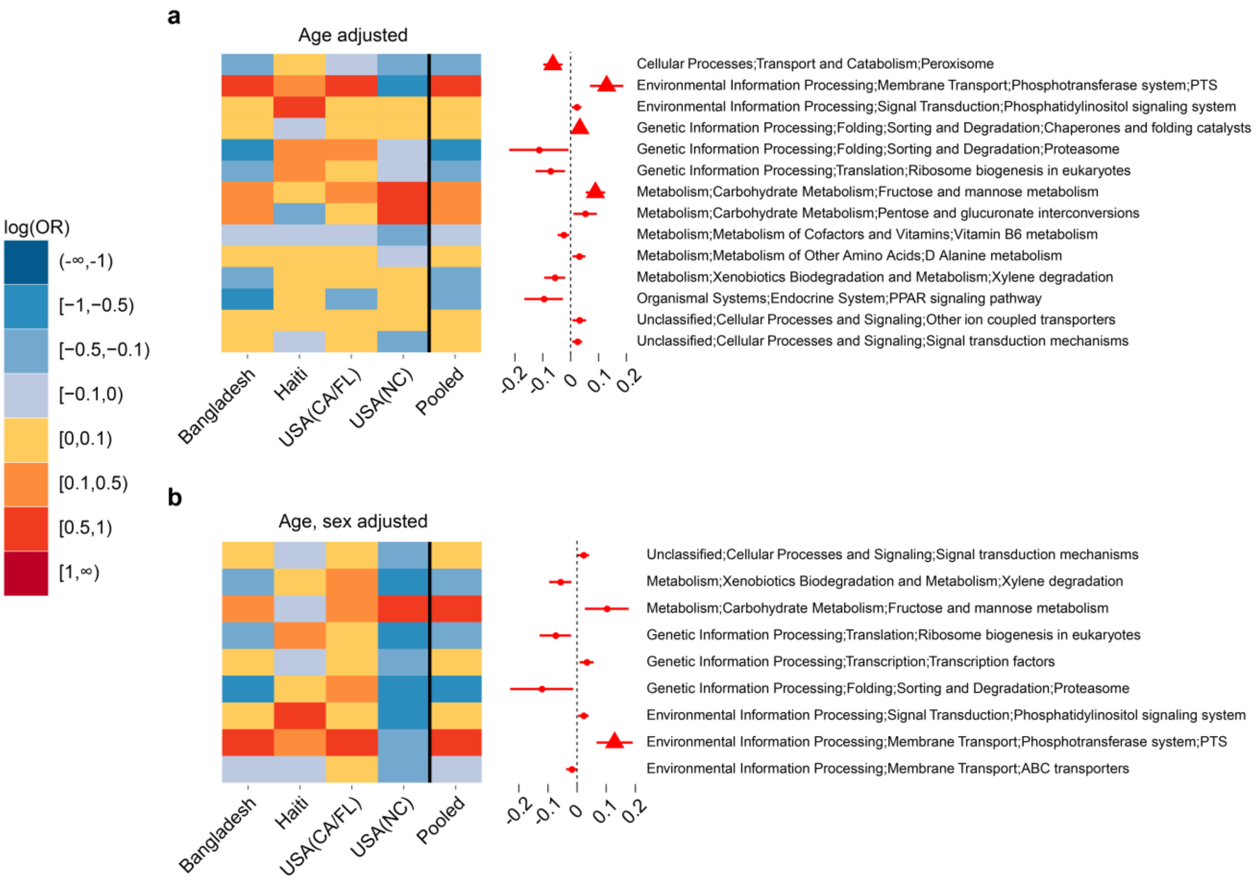

a. Non-exclusive breastfeeding (non-EBF) vs. EBF adjusting for infant age.

b. Non-EBF vs. EBF adjusting for infant age and sex.

The analysis was done in a subset of four studies with available data on infant sex (Bangladesh, Haiti, USA [California-Florida], USA [North Carolina]).

The figures show heatmap of log(odds ratio) (log(OR)) of relative abundances of gut microbial KEGG pathways at level 3 between non-exclusively breastfed (non-EBF) vs. exclusively breastfed (EBF) infants for each study and forest plot of pooled estimates of four studies with 95% confidence intervals (95% CI).

All log(OR) estimates of each pathway from each study were from Generalized Additive Models for Location Scale and Shape (GAMLSS) with zero inflated beta family (BEZI) and were adjusted for infant age at sample collection (a) or adjusted for both infant age at sample collection and infant sex (b). Pooled log(OR) estimates and 95% CI (forest plot) were from random effect meta-analysis models based on the adjusted log(OR) estimates and corresponding standard errors of all included studies. Pooled log(OR) estimates with pooled p-values<0.05 are in red and those with false discovery rate (FDR) adjusted pooled p-values <0.1 are in triangle shape. Only pathways with FDR adjusted pooled p-value <0.1 are shown.

KEGG: Kyoto Encyclopedia of Genes and Genomes; USA: United States of America; CA: California; FL: Florida; NC: North Carolina.

Supplementary Figure 18. Longer duration of exclusive breastfeeding is associated with reduced effects of diarrhea at the time of stool sample collection on gut microbial diversity of infants from 6 months to 2 years of age.

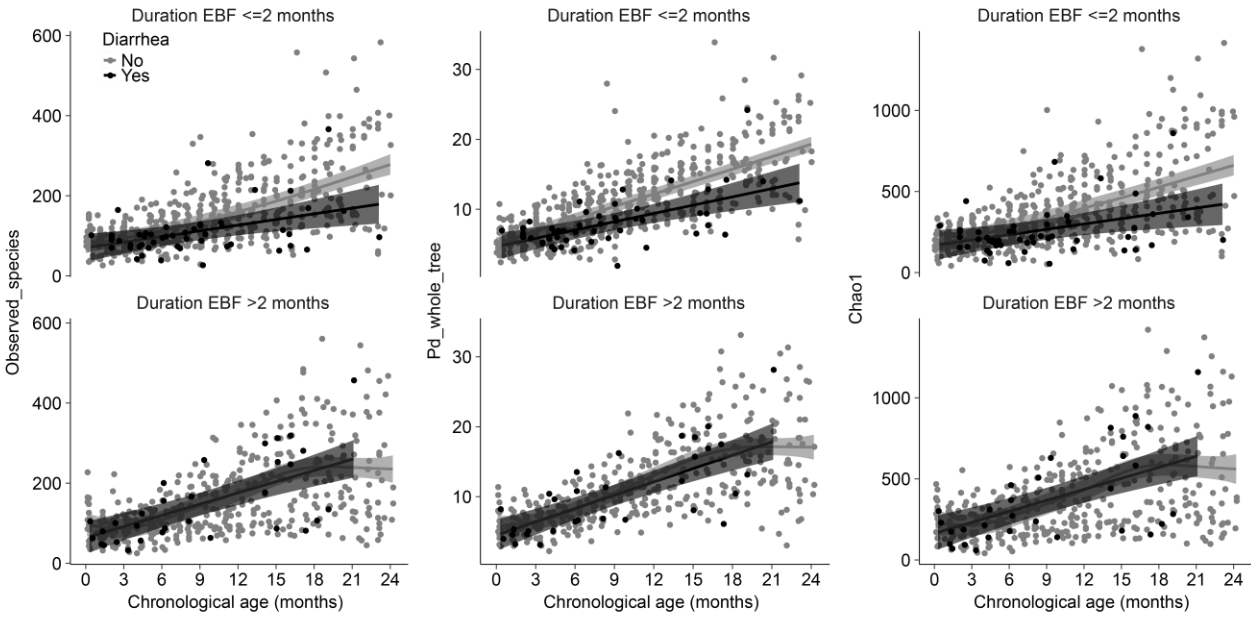

Alpha diversity indexes: Observed species, Phylogenic diversity whole tree (PD_whole_tree) and Chao1 are shown.

Fitted lines and 95% confidence intervals (95% CI) were from Generalized Additive Mixed models (GAMM).

Supplementary Notes

Supplementary Note 1. List of Canadian Healthy Infant Longitudinal Development (CHILD) study investigators.

Subbarao P (Director), The Hospital for Sick Children & University of Toronto; Turvey SE (co-Director), University of British Columbia; Sears MR, (Founding Director), McMaster University; Anand SS, McMaster University; Becker AB, University of Manitoba; Befus AD, University of Alberta; Brauer M, University of British Columbia; Brook JR, University of Toronto; Chen E, Northwestern University, Chicago; Cyr MM, McMaster University; Daley D, University of British Columbia; Dell SD, The Hospital for Sick Children & University of Toronto; Denburg JA, McMaster University; Duan QL, Queen's University; Eiwegger T, The Hospital for Sick Children & University of Toronto; Grasemann H, The Hospital for Sick Children & University of Toronto; HayGlass K, University of Manitoba; Hegele RG, The Hospital for Sick Children & University of Toronto; Holness DL, University of Toronto; Hystad P, Oregon State University; Kobor M, University of British Columbia; Kollmann TR, University of British Columbia; Laprise C, Université du Québec à Chicoutimi; Lou WYW, University of Toronto; Macri J, McMaster University; Mandhane PJ, University of Alberta; Miller G, Northwestern University, Chicago; Moraes TJ, The Hospital for Sick Children & University of Toronto; Paré P, University of British Columbia; Ramsey C, University of Manitoba; Ratjen F, The Hospital for Sick Children & University of Toronto; Sandford A, University of British Columbia; Scott JA, University of Toronto; Scott J, University of Toronto; Silverman F, University of Toronto; Simons E, University of Manitoba; Takaro T, Simon Fraser University; Tebbutt SJ, University of British Columbia; To T, The Hospital for Sick Children & University of Toronto.
